# A computational exploration of global and temporal dynamics of selection pressure on HIV-1 Vif polymorphism

**DOI:** 10.1016/j.virusres.2024.199323

**Published:** 2024-01-19

**Authors:** Md Sakil Arman, Md Zafrul Hasan

**Affiliations:** Department of Biochemistry and Molecular Biology, Shahjalal University of Science and Technology, Sylhet 3114, Bangladesh

**Keywords:** HIV-1 Vif, APOBEC3, Immune pressure, dn/ds, Entropy, Mutational dynamics, Evolutionary trend, Mutational plasticity, Protein stability, HLA-restricted CD8+ T cell

## Abstract

Virion infectivity factor (Vif), an accessory protein of HIV-1 (human immunodeficiency virus type 1), antagonizes host APOBEC3 protein (apolipoprotein B mRNA editing enzyme, catalytic polypeptide 3) or A3 via proteasomal degradation, facilitating viral replication. HLA (Human leukocyte antigens) alleles, host restriction factors, and error-prone reverse transcription contribute to the global polymorphic dynamics of HIV, impacting effective vaccine design. Our computational analysis of over 50,000 HIV-1 M *vif* sequences from the Los Alamos National Laboratory (LANL) database (1998–2021) revealed positive selection pressure on the *vif* gene (nonsynonymous to synonymous ratio, dn/ds=1.58) and an average entropy score of 0.372 in protein level. Interestingly, over the years (1998–2021), a decreasing trend of dn/ds (1.68 to 1.47) and an increasing trend of entropy (0.309 to 0.399) was observed. The predicted mutational frequency against Vif consensus sequence decreased over time (slope = -0.00024, *p* < 0.0001). Sequence conservation was observed in Vif functional motifs F1, F2, F3, G, BC box, and CBF β binding region, while variability was observed mainly in N- and C- terminal and Zinc finger region, which were dominantly under immune pressure by host HLA-I-restricted CD8+ T cell. Computational analysis of ∆∆G^stability^ through protein stability prediction tools suggested that missense mutation may affect Vif stability, especially in the Vif-A3 binding interface. Notably, mutations R17K and Y44F in F1 and G box were predicted to destabilize the Vif-A3 binding interface by altering bond formations with adjacent amino acids. Therefore, our analysis demonstrates Vif adaptation with host physiology by maintaining sequence conservation, especially in A3 interacting functional motifs, highlighting important therapeutic candidate regions of Vif against HIV-1 infections.

## Highlights

• Analyzed over 50,000 Vif sequences of HIV-1 M group from global circulating strains.
• Revealed Vif evolutionary dynamics under host-mediated selection pressure.
• Vif is under positive selection, with contrasting trends in dn/ds and entropy.
• Vif functional motifs are conserved, offering targets for universal HIV-1 vaccines.
• Vif showed mutational plasticity in binding sites, crucial for HIV-1 adaptability.

## Graphical abstract

**Figure:**
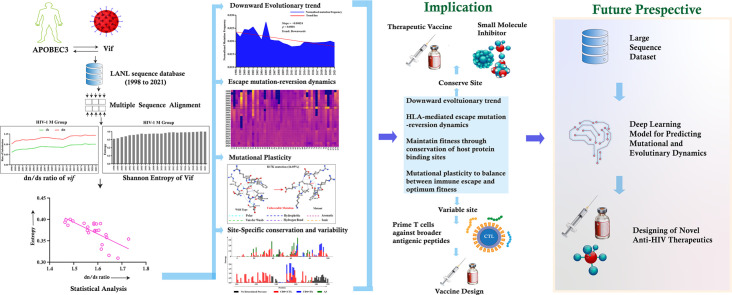
Graphical abstract: Graphical Overview of HIV-1 Vif's Evolutionary Dynamics. Our extensive analysis of over 50,000 HIV-1 Vif sequences (1998-2021) from the LANL database highlights the mutational dynamics and adaptive strategies of Vif, emphasizing an equilibrium between sequence conservation and immune escape. This signifies the potential of Vif as a target candidate for universal HIV vaccines and therapeutic strategies. Looking forward, the application of deep learning for projecting Vif's mutational and evolutionary trajectories may significantly shift HIV's treatment design, paving the way for innovative approaches to combating the virus.

## 1 Introduction

The arms race between host immune responses and viral evasion strategies significantly shapes the evolutionary dynamics of human immunodeficiency virus type 1 (HIV-1), contributing to its sequence polymorphism in global circulating strains (Mwimanzi et al., 2010). The genetic variability and mutational dynamics of HIV-1 due to host immune pressure, error-prone reverse transcription, and antiretroviral therapy (ART) present a major obstacle for designing universal and durable vaccines (Haynes et al., 2023). Among the host immune responses, HLA-restricted cytotoxic T lymphocytes (CTLs) or CD8+ T cell is recognized as the primary immune force of HIV-1 evolution (Carlson and Brumme, 2008), while the viruses also mutate to escape or adapt from other forces (e.g., CD4+ T cells, NK, B cells, and host restriction factors) to maintain a balance of immune evasion with the cost of viral fitness (Jost and Altfeld, 2012; Erdmann et al., 2015; Mujib et al., 2017; Harris et al., 2003).

Among the myriad factors contributing to this complex interplay of HIV-1 sequence evolution, the accessory protein, virion infectivity factor (Vif), plays a pivotal role in neutralizing host innate defenses, particularly by antagonizing host restriction factor APOBEC3 (A3) proteins (Sheehy et al., 2002). Unlike other cellular restriction factors, A3 proteins contribute to viral evolution by inducing G-to-A hypermutations in the viral genome, compromising viral fitness and replicability (Sheehy et al., 2002). Hypermutated HIV genomes generate misfolded proteins and antigenic peptides through proteasomal processing, which is presented on the cell surface and enhances cytotoxic T lymphocyte (CTL) recognition of infected cells. This phenomenon signifies the synergistic interaction between innate and adaptive immunity in HIV-1 restriction (Casartelli et al., 18). Vif strategically counters this immune defense mechanism by selectively degrading human A3 proteins via the ubiquitin/proteasome-dependent pathway, thereby ensuring viral survival and pathogenesis (Yu et al., 2003).

It is documented that distinct motifs of Vif serve as interaction sites for various host A3 proteins (e.g., A3F, A3G, A3H, A3D, and stable A3H), allowing for targeted antagonism (Hultquist et al., 2011). For Vif-A3F interaction, F1 (^14^DRMR^17^), F2 (^74^TGERxW^79^), and F3 box (^171^EDRWN^175^) served as functional interaction sites (He et al., 2008; Russell et al., 2009; ; Dang et al., 2010), while the G box motif, ^40^YRHHY^44^, is pivotal for Vif-A3G binding (Russell et al., 2009; Mehle et al., 2007). The motifs of Vif, which interact with both A3G and A3F, include ^21^WxSLVK^26^, FG box ^55^VxIPLx4-5LxΦx2YWxL^72^ and ^69^YxxL^72^ (Dang et al., 2009; Chen et al., 2009; Pery et al., 2009). Other A3 family members, such as A3C, share common binding sites with Vif residues ^12^QVDRMR^17^, which overlaps with the Vif-A3F binding surface (Pery et al., 2009). Vif interaction with A3H is mainly facilitated by Vif amino acids located at positions 39 and 48 (Ooms et al., 2013a, 2013b). Additionally, the amino acids from positions 60 to 63 (GDAK) and the motif ^69^YxxL^72^ also play roles in this interaction (Refsland et al., 2014). Interstingly, amino acid phenylalanine or tyrosine at position 39 in Vif is crucial for A3H degradation, while a mutation at position 63 diminishes Vif's counteractive efficacy against A3H by two-fold (Ooms et al., 2013a). Vif-mediated proteasomal degradation of A3 proteins is dependent on the formation of an E3-ubiquitin ligase complex, which includes host protein Cullin 5 (CUL5), Elongin B and C (ELOB/ ELOC), Ring-Box 2 (RBX2), and Core-binding factor subunit beta (CBFβ) (Yu et al., 2003; Zhang et al., 2012). Although CBFβ lacks ubiquitylation activity, it stabilizes the complex by engaging with Vif via specific CBFβ-binding motifs, namely ^84^GxSIEW^89^ and ^102^LADQLI^107^ (Wang et al., 2014; Matsui et al., 2014). Two motifs of Vif take part in binding with the E3 ligase complex: BC box motif (^144^SLQYLA^149^), which mimics human SOCS, binds with Elongin C (Yu et al., 2004) while zinc-binding motif (^108^Hx_5_Cx_17_−18Cx_3_−5H^139^) interact with CUL5 (Mehle et al., 2006). The CUL5-Vif-CBFβ complex interacts with ARIH2, essential for the monoubiquitination of A3 proteins, which transfers ubiquitin directly to the A3 targets and can promote polyubiquitination by priming other cellular substrates (Hüttenhain et al., 2019). The ELOB/ELOC heterodimer in the E3 ubiquitin ligase complex facilitates the polyubiquitination of designated proteins, where CUL5 functions as the architectural scaffold, crucial for targeted ubiquitination. The amino acid side chain in ^149^A of the BC box and the amino acid residues at ^120^IRxxL^124^ of the Zinc finger region are the primary mediators for binding with ELOB and CUL5, respectively (Wang et al., 2014). Cullin 5 interacts with human RING finger protein 2 (RBX2), which subsequently interacts with the E2 ubiquitin-conjugating enzyme. This resulted in K48-linked polyubiquitination across various lysine sites on the A3 enzyme, marking it for proteasomal degradation (Jäger et al., 2012).

Besides its primary function of A3 antagonism, Vif is involved in the late phase of the viral life cycle: virion assembly and release (Batisse et al., 2012), especially in virion maturation (Akari et al., 2004). Moreover, the N-terminal domain of Vif interacts with viral RNA, which is essential for its inclusion in newly formed viral particles (Batisse et al., 2012). Mutagenesis studies also suggested the potential roles of Vif in maintaining the structural integrity of the viral core (Batisse et al., 2012). Vif also plays a pivotal role in the cell cycle arrest of T cell lines by proteasomal degradation of protein phosphatase 2A (PP2A) regulators using the similar host cell protein that polyubiquitinates A3 proteins (Salamango et al., 2019; Greenwood et al., 2016).

The multifaceted role of Vif in viral pathogenesis offers potential therapeutic avenues for designing different treatment strategies targeting Vif antagonistic functions. Therefore, uncovering the sequence polymorphism and evolutionary dynamics of Vif is extremely important for identifying new targets against HIV-1. Previous studies suggest that HIV-1′s global sequence diversity in response to immune pressures, especially CTL, generally follows predictable patterns, which could be deduced by contemporary bioinformatics tools (Carlson et al., 2008). The variability of different HIV proteins due to host-mediated selection pressure has been investigated for Gag, Pol, RT, Nef, Vpu, and Tat (Hasan et al., 2012, 2020; Mwimanzi et al., 2011; Brumme et al., 2007, 2009), while the temporal and global dynamics of sequence variability of the Vif is an area yet to be explored. Therefore, this study aims to computationally explore the impact of selection pressures on Vif using 24 years of global sequence data (1998–2021) from the Los Alamos National Laboratory (LANL) HIV sequence database. This fundamental research work could elucidate the mutational dynamics of Vif and pave the way for future research on novel HIV therapeutics.

## 2 Materials and methods

### 2.1 Sequence retrieval and dataset creation for HIV-1 *vif*

A total of 51,608 raw sequences of the *vif* gene from HIV-1 M group, known for its high prevalence and role in the global pandemic (Robertson et al., 2000), along with recombinant forms, were retrieved from the Los Alamos National Laboratory (LANL) sequence database for the period 1998 to 2021. Raw nucleotide sequences were arranged chronologically and categorized based on subtypical variation (e.g., A, B, AE, and others) using the Biopython v1.81 package (Cock et al., 2009) in the Python programming language. They were further classified into primary or pure subtypes, circulating recombinant forms (CRFs), and unique recombinant forms (URFs), based on the information from the LANL database and relevant literatures. Reference consensus sequences of *vif* for different HIV-1 subtypes (Linchangco et al., 2022) were also retrieved and aligned with the autologous sequences using the muscle algorithm in MEGA11 (Kumar et al., 2018). The Muscle algorithm is noted for its accuracy and speed, particularly when aligning extensive sequence data (Edgar, 2004). Besides, the gene sequences were refined by stripping out the gaps and incomplete sequences, followed by translation into protein using standard amino acid codes in MEGA11.

### 2.2 Determination of selection pressure on *vif*

To quantify the selection pressure on HIV-1 *vif* genes, the nonsynonymous (dn) to synonymous (ds) substitution ratio (dn/ds) was determined by SNAP v2.1.1 from the LANL database (Korber, 2002). This tool used multiple aligned nucleotide sequences as queries and calculated the dn and ds values for 192 codons of *vif*. The dn/ds ratio serves as an indicator of selection pressure in which dn/ds ≥ 1 indicates positive or diversifying selection, favoring nonsynonymous substitutions as the virus alters amino acids to evade host defense mechanisms. Conversely, a dn/ds < 1 denotes purifying or negative selection, reflecting a preference for synonymous substitutions and signifying viral adaptation to host immune systems by retaining specific characters optimal for its function within the host physiological environment.

### 2.3 Amino acid variability of Vif protein

Shannon entropy score was used to measure the amino acid variability at each position of the Vif to decipher accumulated mutations over time. A higher entropy value implies functional adaptability and responsiveness to physiological changes, whereas a lower value denotes the conservation of critical functional motifs or structural elements. To see the amino acid variability of Vif, Entropy-One tool in the LANL database was utilized that used aligned amino acid sequences as input to calculate entropy scores for each amino acid position of the protein. These entropy values were calculated for Vif sequences across multiple analytical contexts, such as calculating variability of a single amino acid position by aggregating sequences from 1998 to 2021 or categorizing sequences into subtypical classifications such as Pure, CRFs, and URFs, which allowed for a multifaceted examination amino acid variability.

### 2.4 Sequence conservation of Vif functional motifs

Sequence logos visually represent amino or nucleic acid variability in multiple sequence alignments, showcasing dominant/over-representing mutations. The vertical dimension of a stack in the sequence logos illustrates sequence conservation, while the height of individual symbols within a stack corresponds to the relative frequency of each amino acid at that position. For Vif functional motifs, sequence logos were generated to visually represent amino acid frequency at specific positions using the WebLogo (Crooks et al., 2004) and Matplotlib (Hunter, 2007) packages within a custom Python script that processes the entire Vif sequence dataset spanning from 1998 to 2021. The motif range was predefined in the script to specify the amino acid positions. Additionally, amino acids were color-coded based on their chemical properties to facilitate visual interpretation of the sequence logos.

### 2.5 Selective pressure on Vif variability and immunogenicity

CTL/CD8+, T helper/CD4+ cells, and the host restriction factors play pivotal roles in HIV-1 sequence variation. To examine the host immune-mediated selection pressure on each amino acid position of Vif, epitope information was extracted from the HIV molecular immunology database corresponding to CTL/CD8+ and T helper/CD4+ restricted positions. The signature amino acids that interact specifically with the A3 protein family, as mentioned in various literatures, were hypothesized to be under Darwinian selection from the A3 protein. The immune pressure and corresponding amino acid variability were depicted in a bar diagram generated through a custom Python script. In this diagram, the height of each bar represents the Shannon entropy score, indicating the variability at each position, while the color provides a qualitative depiction of the diverse immune pressures on Vif residues mediated by CD8+ T cell, CD4+ T cell, or A3 protein. Additionally, epitope density maps for CD8+ and CD4+ T cells and antibodies were plotted against Vif amino acid residues. Fisher's exact test determined the association between amino acids under selection pressure and the incidence of CD8+ T cell-induced escape mutations, as per LANL database records. MHC binding affinities of CD8+ T cell epitopes and their variants were analyzed using MHCflurry 2.0 (O'Donnell et al., 2020, 2018), where a higher score indicates a weaker binding to the MHC molecule, potentially reflecting an adaptive mechanism for immune escape.

### 2.6 Geographical distribution of dominating mutation of Vif functional motifs

The geographical information for each Vif sequence was extracted from the corresponding sequence header (for example, B.AR.00.85323FL 2000.KY968402), where country names were encoded using ISO 3166-1 alpha-2 codes (e.g., AR represents Argentina). The sequence dataset was processed using Python scripts, which counted the total number of sequences and determined the frequency of dominating or over-representing mutations in each country. The frequency of these mutations of the Vif functional motif was used as a color scale to portray the dynamics of mutations across different countries. Additionally, the most prevalent mutation for the countries was overlayed on the world map. This visualization was achieved using the GeoPandas package in Python (Jordahl et al., 2020).

### 2.7 Mutational landscape and evolutionary dynamics of HIV-1 Vif

The mutational dynamics and evolutionary patterns of the Vif protein from 1998 to 2021 were investigated against a consensus sequence derived from a dataset of over 50,000 sequences from LANL, utilizing Biopython (Cock et al., 2009), Matplotlib (Hunter, 2007), and additional packages. Given the disparate sequence counts per annum, the yearly mutation frequency at each position of Vif was computed by dividing the mutation count by the total sequences for that year. To account for the varying sequence number and low-frequency values, the normalized mutation frequency for each position and year was adjusted further by dividing by the total mutation at that position over all years and then scaling by a factor of 1000. This normalization and scaling process made the mutation frequencies more interpretable, allowing for a better understanding of the temporal dynamics of amino acid frequency in the Vif protein. Subsequent time series analysis on the yearly average mutational frequency delineated the pattern of Vif evolution over time. Additionally, the overall mutational landscape of Vif was ascertained for each of the 192 amino acids through a custom Python script.

### 2.8 The effect of point mutation on Vif stability

Nonsynonymous point mutations can alter a protein's three-dimensional structure by affecting its conformation, which is related to stability and functionality (Dobson et al., 1998). To assess the impact of dominating mutations, four protein stability prediction tools were employed: DDMut (Zhou et al., 2023), DynaMut2 (Rodrigues et al., 2021), PremPS (Chen et al., 2020), and DDGun3D (Montanucci et al., 2022), each utilizing distinct machine learning algorithms to calculate protein stability against missense mutations.

Dominating mutations were identified from the compiled sequence dataset based on their frequency at each position along the ORF, automated through a custom Python script. The dominant mutation at each position was defined as either the second most common amino acid or the mutation with the highest frequency, aside from the wild type, which is represented by the consensus strain. A 3D structure of unbound HIV-1 Vif protein was generated using AlphaFold2 in Google Colab (Mirdita et al., 2022, Jumper et al., 2021) based on the consensus sequence. This structure served as a query in the stability prediction tools to determine the effect of missense mutations on Vif functional motifs. DynaMut2 and DDMut estimate ΔΔG^stability^, a specialized metric that quantifies the differential change in Gibbs free energy between wild-type and mutant protein structures upon folding, where a positive value indicates a stabilizing or favorable mutation, while a negative value denotes a destabilizing or unfavorable mutation. The results from the rest of the tools were adapted to match this convention.

### 2.9 Statistical analysis and data processing

Statistical analyses were performed utilizing GraphPad Prism 9 (GraphPad Software, Boston, MA, USA), which included Spearman correlation, two-way ANOVA, Exact Fisher's, and Mann-Whitney U tests in light of the non-normal data distributions and unequal variance. The threshold for statistical significance was set at *p* < 0.05, and significant findings are denoted by an asterisk (*). Data processing and analysis were conducted through custom Python scripts generated with the aid of GPT-4. Adobe Illustrator was used to enhance the quality of the figure derived from the analysis.

## 3 Results

### 3.1 Compilation of Vif sequence dataset

The raw sequences of HIV-1 *vif* from 1998 to 2021 were retrieved from LANL, a global HIV sequence repository. The sequences were sorted into distinct subtypes (*n* = 72) for further analysis based on availability and ubiquity. Among these, ten were identified as pure subtypes (*A, B, C, D, F, G, H, J, K, and L*), while the rest were divided into 22 CRFs (*0107, 0108, 01A, 01B, 0206, 02A, 02B,* 02*G, 06A, 06B, AB, AD, AE, AF, AG, BC, BF, BG, CD, DF, 07B, and 01C*) and 44 URFs (*0102, 0102A, 01ADF, 01AF, 01AG, 01BC, 01FG, 0209, 0213, 0222, 0225, 0263, 02AF, 02AG, 02BF, 02BG, 02C, 02D, 02F, 02FG, 02GK, 02H, 14F, 32A,* 32*G, 50B, AC, ACD, ACG, ADG, AFG, AGH, AH, AK, BCF, BCG, ABD, DG, BD, and CF*), based on literature review and LANL database. CRFs (Circulating Recombinant forms) are generally circulating strains in the global populations identified in multiple individuals who are not epidemiologically linked, whereas URFs (Unique Recombinant forms) have been identified in a single individual or epidemiologically linked individuals, are not found in circulating in the broader population (Robertson et al., 2000). The final dataset encompassed 51,608 selected nucleotide sequences of the *vif* gene, sourced from circulating HIV strains across 92 countries (Supplementary Table ST1). The nucleotide sequences were translated into 50,788 corresponding protein sequences, and 820 sequences were removed because of partial and incomplete data sets.

### 3.2 Host-mediated selection pressure on *vif* gene of HIV-1 group M

**Fig. 1:**
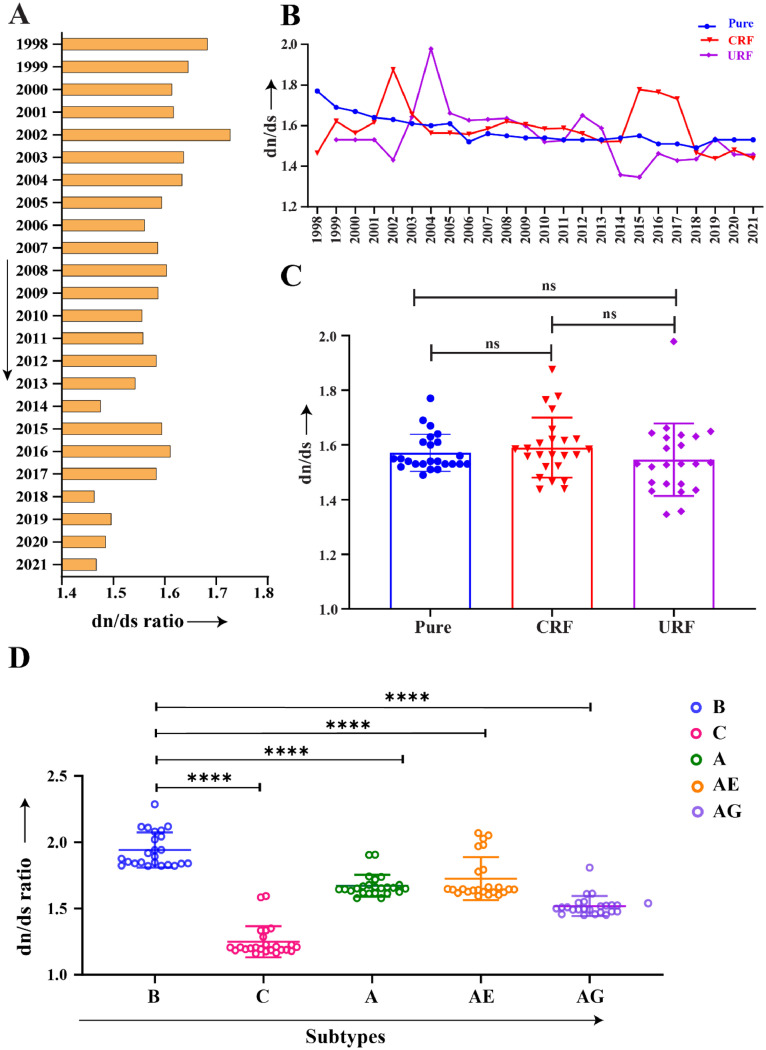
Host-mediated selection pressure on *vif* gene. (A) Average dn/ds ratio of the *vif* from HIV-1 M group using nucleotide sequences from LANL database (*n* = 51,608), spanning 1998 to 2021. (B) Selection pressure on *vif* among Pure subtypes (*n* = 36,502 seq), CRFs (*n* = 12,868 seq), and URFs (*n* = 2238 seq) from 1998 to 2021. (C) Statistical analysis suggested the difference of dn/ds ratio among the subtypical classifications of the sequence (Pure, CRFs and URFs) were not significant (*p* > 0.05). (D) Temporal comparison of the average dn/ds ratio among the global prevalent subtypes (B, C, A, AE, and AG) revealed a distinct and statistically significant pattern over the same period.

The overall dn/ds ratio for *vif* gene was 1.58, indicating a current positive selection pressure. Interestingly, temporal analysis of dn/ds ratio revealed a fluctuating yet declining trend where the value was 1.68 and 1.47 for 1998 and 2021, respectively (Fig. 1A). However, a complex pattern emerged when we categorized *vif* sequences into pure, CRFs, and URFs. Pure subtypes showed a gradual decrease in overall dn/ds ratio over time (max: 1.77, min: 1.49, avg: 1.57) while the CRFs (max:1.88, min: 1.44, avg: 1.59) and URFs (max: 1.98, min: 1.35, avg: 1.55) exhibited significant fluctuation patterns, which may be attributed to their genetic complexity and the timing of their emergence over the course of infection (Fig. 1B). This pronounced difference in dn/ds pattern among the pure subtypes, CRFs, and URFs was not statistically significant (*p* > 0.05), determined by the Mann-Whitney U test, based on data distribution and unequal variance (Fig. 1C). Remarkably, in a global and temporal frame (1998–2021), significant variation of selection pressure was observed on the *vif* gene among the dominating HIV-1 subtypes (Fig. 1D).

Subtype B, predominantly sequenced from regions of the Americas and European Regions, differed significantly (*p* < 0.0001) from other globally prevalent subtypes C, A, AE, and AG (Williams et al., 2023). The variations in selection pressure across different subtypes may be a reflection of the disparities in host HLA loci across distinct global regions.

### 3.3 Amino acid variability of HIV-1 Vif protein

**Fig. 2:**
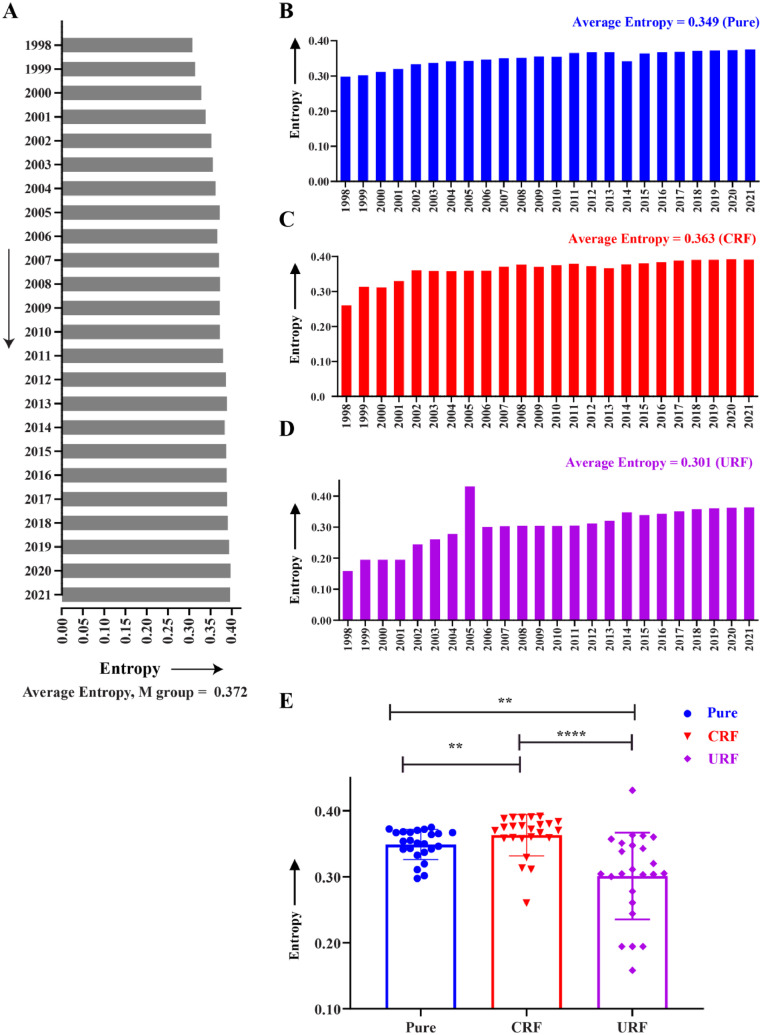
Amino acid variability of HIV-1 Vif protein. (A) Average Shannon entropy score of Vif from 1998 to 2021 based on global circulating strains (*n* = 50,788 seq). Temporal analysis of average entropy score upon classification of the sequences into (B) pure (*n* = 35,702 seq), (C) CRFs (*n* = 12,841 seq), and (D) URFs (*n* = 2245 seq). (E) Statistical analysis was performed to compare the average entropy scores among pure, CRFs, and URFs subtypes, revealing significant variation.

A temporal analysis of the amino acid variability of the HIV-1 Vif protein from 1998 to 2021 was conducted to examine the effects of selection pressure at the protein level. We employed Shannon entropy as a metric, which quantifies the uncertainty or variability in a dataset. In this study, higher entropy scores indicate greater amino acid variability, reflecting evolutionary pressures. The average entropy score for the Vif protein among circulating HIV-1 strains (*n* = 50,788 seq) was 0.372. The entropy scores of Vif showed a gradual increase over the year, with a minimum of 0.309 in 1998 and a maximum of 0.4 in 2020 (Fig. 2A). The same increasing trend was observed when we divided the sequences into pure subtypes, CRFs, and URFs, suggesting a consistent amino acid variability over time. The URFs (0.158 to 0.363, avg = 0.301), however, showed a sharp increase in entropy score over the year, compared with pure subtypes (0.298 to 0.375, avg = 0.349) and CRFs (0.260 to 0.391, avg = 0.363), with a notable surge in entropy in 2005, potentially indicative of the emergence of new subtypes (Fig. 2B–D). Statistical analyses also highlighted significant differences (*p* < 0.05) in entropy trends between these groups over time (Fig. 2E).

**Fig. 3:**
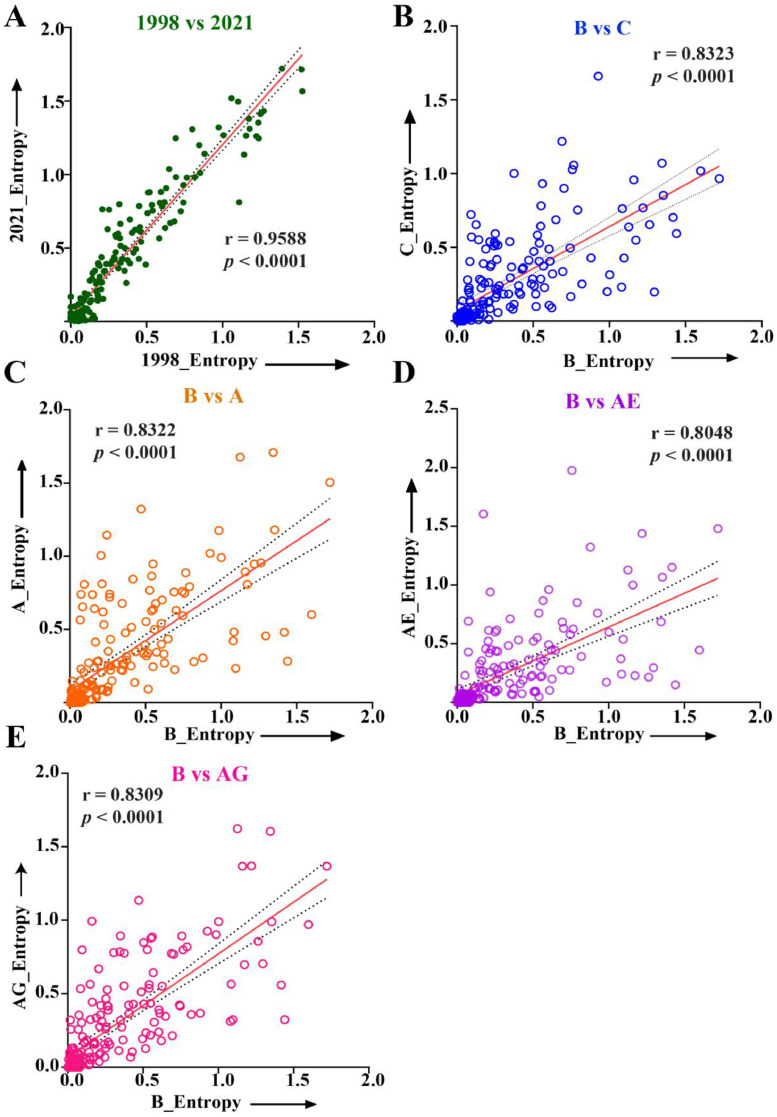
Spearman's rank-order correlation to ascertain amino acid variation. (A)Variation within the M group between 1998 and 2021. Subtypical variation from 1998 to 2021 between (B) B and C, (C) B and A, (D) B and AE, and (E) B and AG, where 'r' represents the correlation coefficient.

In addition, the Spearman correlation was used to investigate the variations of each 192 amino acid position of Vif within the M group between 1998 and 2021 (Fig. 3A). The analysis indicated a strong positive correlation (*r* = 0.9588, and *p* < 0.0001) and a consistent trend in amino acid variability within the M group over this time frame.

Moreover, analysis of amino acid variations among globally prevalent subtypes B, C, A, AE and AG yielded a statistically significant variation (*p* < 0.05), with correlation coefficients (r) approaching 1, indicating that amino acid variability of subtype B closely aligns with changes in the other subtypes (Fig. 3B–E). These results emphasize the strong positive relationship between the prevalent subtypes, revealing a consistent trend in the global amino acid variability in Vif over time.

### 3.4 Sequence conservation of Vif functional motifs

To effectively visualize and analyze sequence conservation and variability within the Vif functional motifs of the HIV-1 M group, we utilized sequence logos, which depict the relative frequency or conservation of each amino acid at specific positions in a multiple sequence alignment. By utilizing WebLogo (Crooks et al., 2004), we analyzed our sequence dataset of the HIV-1 M group (*n* = 50,788 sequences), identifying conserved residues critical for Vif function and areas of variability, which might reflect adaptive responses to host selection pressures.

**Fig. 4:**
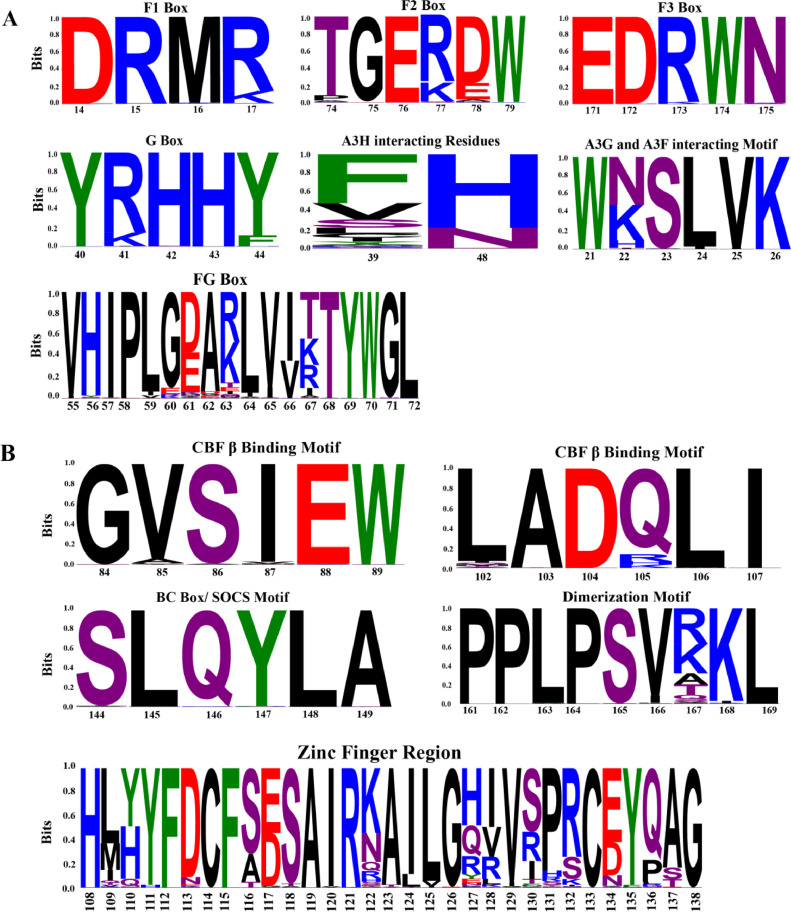
Sequence conservation in Vif functional motifs from 1998 to 2021. (A) Sequence logo of Vif motifs that interact with host A3 proteins. (B) Conservation in C-terminal key motifs that interact with E3 ubiquitin ligase complex. Amino acids are colored by their properties: aromatic (green), basic (blue), acidic (red), polar (purple), and hydrophobic (black).

Our analysis of Vif sequences from 1998 to 2021 revealed that the F1 and F2 box motifs demonstrated sequence conservation but also exhibited notable mutations, specifically R17K, R77K, and D78E (Fig. 4A). The G box motifs showed prominent mutations at positions R41K and Y43F, while signature amino acids at positions 39 and 48 also exhibited over-representing mutation. In contrast, the FG box was highly variable, suggesting differential evolutionary pressure (Fig. 4A).

On the contrary, the CBFβ binding motif was found to be highly conserved, similar to the BC box motif (Fig. 4B) based on analysis of 24 years of sequence data in our study. However, the dimerization motif (PLPPx4L) of Vif displayed considerable variation at position 167. The Cullin 5 interacting Zn finger region exhibited high variability, indicative of potentially differential selection pressures (Fig. 4B).

### 3.5 Geographical patterns of key mutations in Vif functional motifs

**Fig. 5:**
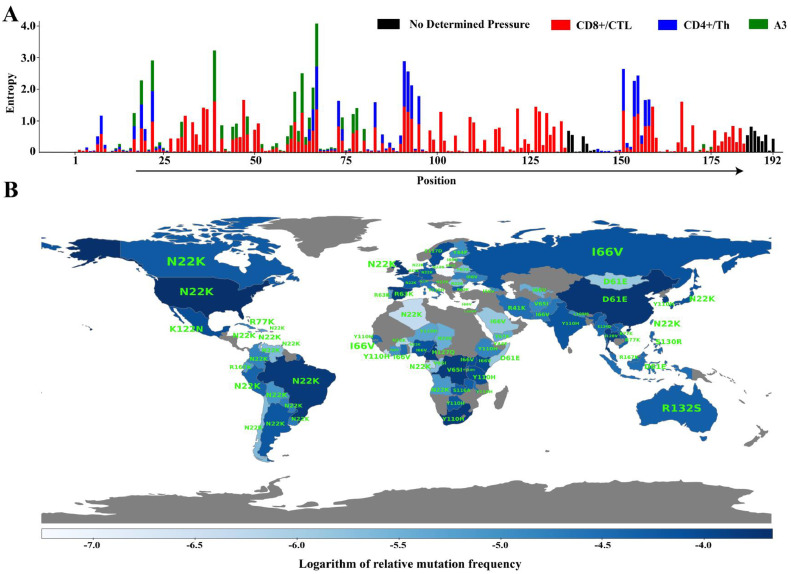
Global variability and mutations in Vif functional motifs, driven by host immune pressure (A) The stacked bar diagram depicts the amino acid variability at each of the 192 positions in Vif due to the selection pressures exerted by CD8+ T cell (red), CD4+ T cell (blue), and A3 proteins (green). (B) Geographical distribution of the mutational frequency and prominent mutations in Vif functional motifs, with relative mutation frequency used as a color scale (deep blue: high frequency and light blue: low frequency) to illustrate how HLA-restricted immune pressures drive the global mutational landscape of Vif.

The influence of host-mediated immune pressure on the global evolution of HIV can be attributed to regional differences in the host HLA expression profile, which force the virus to adopt a spectrum of mutational patterns to escape CTL recognition. In order to investigate this phenomenon in Vif, we mapped the differential immune pressure on Vif residues after retrieving CD8+ and CD4+ T cell epitope information from the Los Alamos Database based on reference HXB2 strains. HLA-restricted immune pressure, especially by CD8+ T cells with higher epitope counts, was associated with greater amino acid variability in Vif, indicative of CTLs' primary role in HIV-1 Vif evolution (Fig. 5A). The key functional motifs, primarily interacting with A3G (40–44, G box), A3F (14–17, F box), and other A3 family proteins, were found to be highly conserved, while variable regions appeared to be influenced by combine immune pressure from CTL and CD4+ T cells along with A3 proteins, highlighting their central role in HIV-1 Vif evolution.

Moreover, to examine the regional disparities in HLA-mediated selection pressure on HIV-1 Vif, an extensive analysis was carried out to identify dominating mutation in Vif functional motifs such as F1 box (14–17), F2 box (74–79), F3 box (171–175), G box (40–44), A3F and A3G interacting residues (21–26), FG box (55–72), CBFβ interacting amino acids (84–89 & 102–107), Zinc finger region (108–139), BC box (144–149), and PPLP motif (161–169) across 92 countries. The relative mutation frequency within each country was calculated and normalized against the total count of such mutations. The normalized frequency was depicted on a world map, facilitating regional comparison. Notably, N22K mutation, associated with A3G and A3F interactions, prevailed in European and American regions, while Y110H mutation, linked to the Zn finger region, was common in Africa (Fig. 5B). This regional variance in mutations may correlate with diverse HLA patterns among human hosts, suggesting distinct global immune pressures on HIV-1 Vif.

### 3.6 Immunogenicity and escape mutation dynamics of HIV-1 Vif

**Fig. 6:**
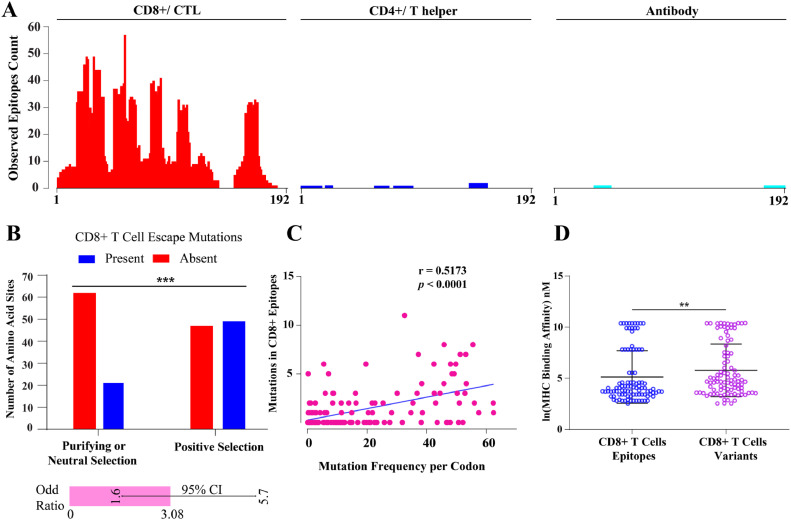
Immunogenicity and escape mechanisms of HIV-1 Vif. (A) Epitope density plot of Vif based on LANL HIV Immunology Database (B) Fisher's exact test on selection pressure and observed CD8+ induced mutations (*n* = 198) across Vif. (C) Spearman correlation of CD8+ escape mutations and codon mutation frequency (D) comparison of MHC binding affinities between CTL epitopes and variants (*n* = 96) by MHCflurry 2.0.

To further elucidate the relationship between Vif immunogenicity and mutational dynamics due to host-mediated selection pressure, we plotted CD8+ and CD4+ T cell as well as antibody epitope density maps of Vif, based on information of clinically observed epitopes deposited in LANL HIV Immunology Database. The high density of CD8+ epitopes compared to CD4+ and antibodies indicated that CD8+ T cells have a greater impact on Vif selection (Fig. 6A).

An Fisher's exact test on selection pressure (positive and negative selection) and the presence of CD8+ T cell-induced escape mutations revealed a statistically significant association (*p* < 0.0007) and an odds ratio of 3.078 (95 % CI: 1.601 to 5.680) (Fig. 6B). This indicates amino acids under positive selection have approximately three times the likelihood of exhibiting escape mutations than those under negative or neutral selection. Furthermore, a Spearman correlation analysis revealed a statistically significant yet a moderate positive correlation (*r* = 0.517, *p* < 0.0001), suggesting an association between higher mutation frequencies and an increased count of CD8+ T cells escape mutations (Fig. 6C), which indicated the moderate immunogenicity of Vif protein. This may also align with the fact that while Vif is subjected to some degree of immune pressure, not all observed mutations are HLA-driven since it is also under functional constraint from A3 proteins. Detailed information on Vif residue under positive selection pressure, affecting Vif-A3 binding with reported CD8+ T cell-induced escape mutation, is presented in Supplementary Table ST2.

To further understand the immunogenicity of the Vif epitope, we compared the MHC binding affinities of CTL epitopes and their corresponding mutant variants using MHCflurry. 2.0. The analysis revealed a statistically significant higher mean binding affinity for variants with escape mutations (Fig. 6D), suggesting that the observed mutations in epitope could be part of an adaptive mechanism, potentially diminishing the efficacy of MHC presentation and facilitating immune escape.

### 3.7 Analysis of Vif mutational landscape and mutational sensitivity

**Fig. 7:**
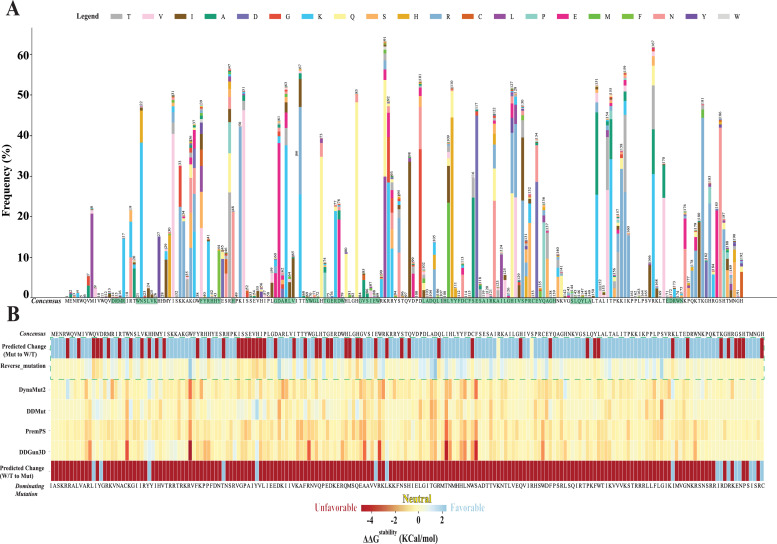
Mutational Landscape and sensitivity of HIV-1 Vif. (A) The mutational landscape of Vif for HIV-1M Group (1998 to 2021) is represented by a stacked bar diagram, with the height corresponding to the specific frequency of mutant amino acids against the consensus sequence on the X-axis. Essential motifs and signature amino acids are shaded in green, with the amino acid position indicated at the top of each bar. (B) The mutational stability of HIV-1 Vif upon dominant missense mutations is depicted in a heatmap, using ΔΔG^stability^ value as a color scale. Negative ΔΔG^stability^ values are depicted in red, signifying unfavorable or destabilizing mutations; light blue indicates stabilizing mutations, while yellow represents neutral or unchanged stability.

The mutational landscape of the Vif protein exhibited significant variation in the temporal and global mutational trends, identifying ∼1538 missense mutations from 1998 to 2021 (*n* = 50, 788 seq) against the consensus sequence. The residues involved in interactions with host A3 proteins demonstrated high conservation with relatively low mutation frequency. Similarly, BC box, which interacts with ELOB/ELOC, and the primary residues of the zinc finger motifs (121 aa to 124 aa), which initially interact with CUL5, revealed sequence conservation with low mutational frequency, whereas the remaining amino acid positions revealed high mutational frequency. (Fig. 7A). This pattern of mutations suggests site-specific dynamics that may influence Vif's evolution over time. Prominent mutations included transitions such as Arginine (R) to Lysine (K), Aspartate (D) to Glutamate (E), and Glutamine (Q) to Histidine (H). Moreover, the impact of these prominent missense mutations on the stability of the Vif three-dimensional structure was predicted by four distinct protein stability prediction tools. Most of the dominant mutations in Vif showed a negative ΔΔG^stability^ and were predicted to be unfavorable or destabilizing, associated with fitness cost (Fig. 7B). Interestingly, hypothetical reverse mutations, representative of reversion to the consensus state, were predicted to be stabilizing (Fig. 7B). This aligns with the notion that ancestral Vif conformations are optimized for binding with host proteins within their functional domains, as mutations might hinder these interactions and thereby cost the virus in the evolutionary arms race. A summary of the impact of point mutations on Vif's functional motifs and signature amino acids is described in Supplementary Table ST3.

### 3.8 Effect of R17K and R41K mutations on Vif-A3F/A3G binding surface

**Fig. 8:**
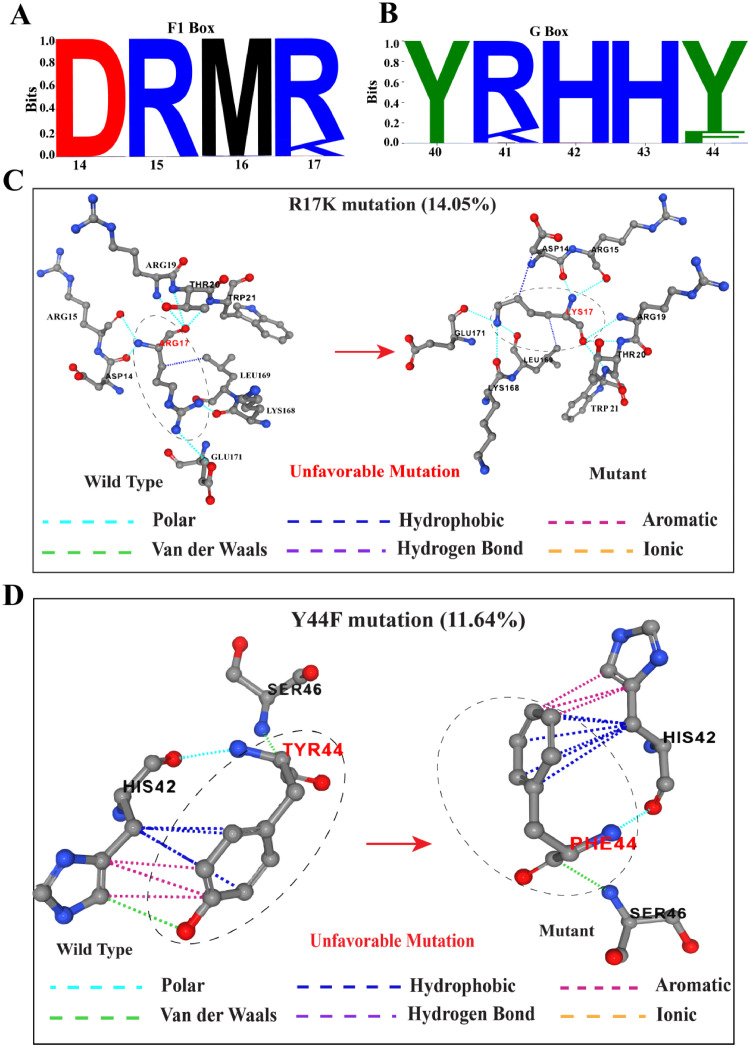
Mutational Sensitivity in Vif-A3 Binding Site. Sequence logo illustrating sequence conservation and mutational frequency of signature amino acids within the (A) F1 box and (B) G box. (C) Comparison of interactions between the amino acid at position 17 and adjacent amino acid across both wild-type and mutant Vif. (D) A similar illustration of interaction for the amino acid at position 44. Interactions are color-coded: cyan for polar, deep blue for hydrophobic, purple for aromatic, orange for ionic, green for van der Waals interactions, and deep violet for hydrogen bonds.

The interaction of Vif with host A3 protein through its functional motifs is crucial for Vif-mediated A3 antagonism. The F1 box motif, known for its interaction with A3F, A3C, and A3D, displayed sequence conservation while featuring R17K mutation (frequency =14.05 %) (Fig. 8A). Similarly, G box motifs, responsible for interacting with A3G, also featured an arginine to lysin mutation (R41K) along with a Y44F mutation (frequency = 11.64 %) (Fig. 8B). Structural comparison between wild type and mutant structure through PremPS tools suggested these observed mutations might compromise the Vif-A3 binding by altering interactions with adjacent amino acids. For instance, the R17K mutant was predicted to form hydrophobic interaction with an aspartate at position 14 rather than polar interactions found in the wild type, which could potentially destabilize the Vif-A3 interface (Fig. 8C). On the contrary, the Y44F mutant did not exhibit the van der Waals interaction with histidine at position 42, a characteristic of the wild type (Fig. 8D). These predicted alterations in the mutants could potentially impact the stability of the binding site and may be detrimental to the virus by hindering Vif-A3 interaction.

### 3.9 Temporal analysis of Vif evolution

To elucidate the evolutionary pattern of Vif, missense mutation frequencies were calculated against the consensus sequence and subsequently normalized to provide an unbiased observation of Vifs' evolutionary dynamics.

**Fig. 9:**
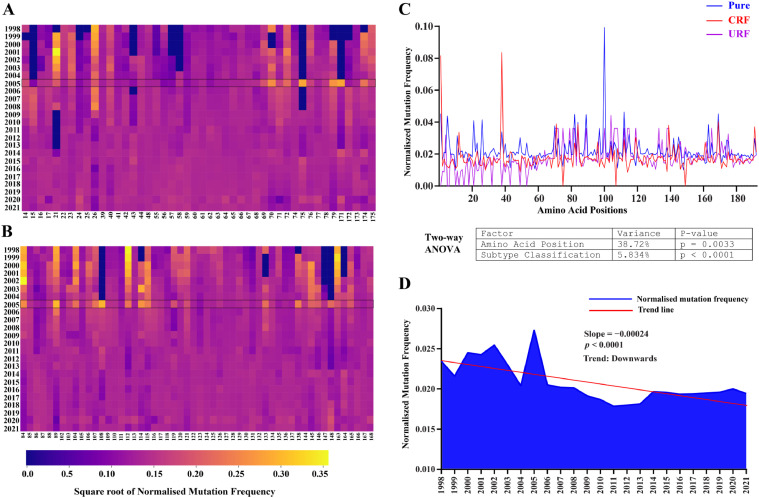
Temporal analysis of Vif mutational and evolutionary Dynamics. Heatmaps display the normalized mutation frequency for (A) A3-interacting residues and (B) E3 ubiquitin ligase interacting signature amino acids by using a plasma color scale based on square root transformation of normalized frequency. Lighter shades denote higher frequencies; deeper shades denote lower frequencies. (C) Normalized mutational frequency of Vif residues from 1998 to 2021 across subtypical groups (pure, CRF, URF). Two-way ANOVA identified the amino acid position and subtypical variation as major contributors to Vif evolutionary dynamics. (D) Time series analysis depicts average mutational frequencies of Vif residues (1–192), fitted in a linear regression model. The trend line's slope represents the rate of change in mutation frequency over time, with negative values indicating a decrease and positive values indicating an increase in mutation rate.

Vif's normalized mutational frequency showed site-specific dynamics and temporal fluctuation for A3 interacting signature amino acids, which peaked in 2005 but declined and stabilized in the following years (Fig. 9A). A similar pattern was observed for the C-terminal signature amino acids involved in Vif-mediated A3 degradation (Fig. 9B). In order to provide a more comprehensive analysis of the mutational dynamics, the mutational frequency of Vif was examined using a two-way ANOVA, which considered amino acid positions and subtypical classifications such as pure subtypes, CRFs, and URFs. The analysis indicated that the amino acid position explained 38.72 % of the overall variation (*p* = 0.0033), suggesting certain positions remain conserved across pure subtypes, CRFs, and URFs, whereas others exhibit greater variability, as indicated by the fluctuation in the mutational frequency (Fig. 9C).

Conversely, 5.834 % of the variance was accounted for by subtypical classification (*p* < 0.0001), suggesting that mutations in Vif are not distributed uniformly throughout the HIV-1 M group. Moreover, implementing linear regression, a time series analysis of the average normalized mutation frequency of Vif amino acids (1–192) from 1998 to 2021 revealed a declining trend line (slope = -0.00024, *p* < 0.001), signifying a marginal yet statistically significant decrease in the mutational frequency of Vif in a non-random occurrence. The observed decrease in mutation frequency could indicate sequence stabilization or optimization, which is consistent with the idea that HIV reverts to ancestral forms for optimal fitness (Fig. 9D).

## 4 Discussion

The global prevalence of HIV-1 infection is intricately linked to its complex mutational dynamics, which is a barrier for designing effective vaccines to date. Furthermore, the regional distribution of genetically varied HIV-1 clades could be linked to various pathogenesis mechanisms, posing considerable obstacles to universal vaccinations (Santoro and Perno, 2013; Dimitrov et al., 2015). The genetic variability of HIV-1 could be attributed to factors such as host-mediated immune pressure, notably HLA-restricted CD8+ T cells, A3-induced hypermutations, and the error-prone nature of reverse transcriptase or recombination events, shaping its mutational landscape and evolutionary trajectory (Sheehy et al., 2002; Brumme et al., 2007; Sewell et al., 2000; Mansky and Temin, 1995). Therefore, a comprehensive understanding of the immune pressures acting on HIV-1 and its mutational and evolutionary dynamics across subtypes is paramount for the innovation of effective and universal formulation against HIV-1 (Moore et al., 2002).

Previous studies have investigated and emphasized the host-mediated immune pressure and the associated sequence variability of various parts of HIV-1, such as Gag, Pol, RT, Nef, Vpu, and Tat (Hasan et al., 2012, 2020; Mwimanzi et al., 2011; Brumme et al., 2007, 2009). Therefore, our current studies focus on the HIV-1 accessory protein Vif, conserved among evolutionary-related lentiviruses (Oberste and Gonda, 1992). Vif in HIV-1 has explicitly evolved to antagonize the host A3 protein family by marking them for proteasomal degradation (Zheng et al., 2005). Moreover, Vif has been identified as a mediating factor for the proteasomal degradation of Protein phosphatase 2A (PP2A) regulators, thereby inducing G2/M cell cycle arrest in multiple T cell lines (Salamango et al., 2019; Greenwood et al., 2016; Marelli et al., 2020). The diverse functional role of Vif makes it a promising candidate for developing novel anti-HIV treatment strategies. This further amplifies the necessity for an in-depth understanding of Vif mutational dynamics and evolutionary trajectories driven by host-pathogen interaction.

Consequently, this study investigates the mutational dynamics and evolutionary patterns of HIV-1 Vif through a series of computational analyses based on retrieved global sequence dataset (*n* > 50,000 seq) from LANL database from 1998 to 2021. Our analytical scope extended to characterizing the Vif mutational landscape due to host immune pressure, mutational plasticity in functional motifs, epitope mapping, and temporal mutation frequencies.

The preliminary analysis of selection pressure, indicated by the dn/ds ratio, revealed that HIV-1 *vif* from the M group was under positive selection pressure, with an average dn/ds of 1.58. However, a declining trend over the years (1998–2021) represents the favoring of synonymous mutations over nonsynonymous ones (Fig. 1A). Upon subtypical classification, pure subtypes showed a gradual decrease in dn/ds ratio, whereas recombinant circulating forms (CRFs and URFs), documented for 29 % of total HIV-1 infection (Williams et al., 2023), showed a complex pattern of dn/ds, which could be attributed to their genetic complexity and time of emergence during recombination event (Fig. 1B). Afterward, Shannon entropy was utilized to determine the degree of amino acid variability in the corresponding Vif protein of the HIV-1 M group; an average entropy score of 0.372 demonstrated its polymorphic nature (Fig. 2A). Remarkably, the entropy scores showed a gradual increase over the year. The same increasing trend of entropy was observed with distinct patterns when we classified the sequences into pure, CRFs, and URFs, suggesting a consistent trend in amino acid variability of Vif over time within global circulating strains of HIV-1 (Fig. 2B–D). Subsequently, comparing entropy scores between globally prevalent subtypes (B, C, A, AG, and AE) yielded a Spearman correlation coefficient (r) close to one, indicating that the variability pattern in subtype B closely aligns with other subtypes (Fig. 3). These results also emphasized a consistent trend in the global amino acid variability in Vif.

To elucidate the interplay between selection pressure, mutational frequency, and Vif immunogenicity, we analyzed CD8+ and CD4+ T cells as well as A3-mediated immune pressure on Vif residues by retrieving epitope information from the Los Alamos Database. Notably, Vif's functional motifs, responsible for interacting with host A3 proteins and E3 ubiquitin ligases complex, showed less amino acid variability (entropy score) compared to other residues under HLA-restricted immune pressures (Fig. 5A). Further, our analysis extended to the plotting of Vif epitope density map, revealing a prominent role of CD8+ T cells in influencing Vif's mutational frequency (Fig. 6A). Fisher's exact test suggested that clinically observed escape mutations are threefold more likely to be associated with amino acids under positive selection pressure (Fig. 6B). However, the moderate positive correlation between mutational frequency and escape mutations suggested that CD8+ T cell-mediated immune pressure is not the sole determinant of the Vif mutational landscape. Most of the Vif residues interacting with A3 proteins were found to be under negative selection, with some notable exceptions (Supplementary Table ST2).

Additionally, the site-specific sequence conservation of Vif functional motifs from 1998 to 2021, as illustrated in sequence logos (Fig. 4), aligned with previous structural studies (Nakashima et al., 2016; Li et al., 2023). The preservation of key amino acids at the binding interfaces for Vif-A3G and Vif-A3F interactions corroborated their functional significance and evolutionary conservation. The differential mutation frequency and prevalence of dominating mutation in these motifs across different geographical regions, e.g., N22K in the American region (Fig. 5B), stressed that differential expression of host HLA profile across continents also shaped HIV-1 evolutionary dynamics (Ragonnet-Cronin et al., 2012; Carlson et al., 2012).

Further, in order to predict the mutational sensitivity of Vif, we utilized DDMut, DynaMut2, PremPS, and DDGun3D to calculate Vif structural stability upon a missense mutation. Since these tools had distinct algorithms for calculating protein stability in mutants, we took a majority rule. The stability heatmap suggested that most of the dominant mutations in Vif could be detrimental to the virus, which may incur fitness costs (Fig. 7B). In contrast, reversion to the consensus forms was predicted to be favorable for HIV-1 Vif, reinforcing the hypothesis that HIV-1 is inclined to revert to ancestral configurations for enhanced fitness, a notion supported by a sequence-based examination of HIV-1 Env (Leslie et al., 2004; Herbeck et al., 2006). The reversion to consensus forms, which hypothetically augments viral protein structural stability as predicted in our study, maybe the rationale for HLA-associated escape mutation-reversion dynamics observed in HIV-1 functional proteins like protease, reverse transcriptase, Vpr, and Nef (Brumme et al., 2007, 2009; Carlson et al., 2012) for minimizing fitness costs.

Analysis of Vif functional motifs, F1 and G box, crucial for A3 bindings, indicated sequence conservation while featuring dominating Arginine-to-Lysine (R17K, R41K) and tyrosine-to-phenylalanine (Y44F) mutations, apparent from the sequence logo (Figs. 8A, B). Our in-silico comparison of wild-type and mutant structures suggested that these mutations may hinder Vif-A3 binding by altering interactions with adjacent amino acids, potentially affecting Vif's antagonistic functions in A3 degradation. Notably, the observed frequency of R17K (14.5 %) and Y44F (11.64 %) mutations in the global sequence database suggests a degree of mutational plasticity in the Vif-A3 binding interface. This might demonstrate the Virus's strategic mutational tolerance by exploiting similar chemical properties of wild-type and mutant amino acids to balance immune escape with minimal fitness costs.

Subsequently, our predictive analysis of the normalized mutational frequency for Vif against consensus sequences suggested fluctuations in the signature amino acids, crucial for interaction with host proteins. Up until 2005, an increase in mutational frequency was noted, which then transitioned to a more dynamic oscillating pattern (Fig. 9A, B). This pattern could mirror the escape mutation-reversion phenomena of HIV-1, observed in the context of HLA-restricted immune pressure (Leslie et al., 2004). Further, time series analysis indicated statistically significant downward evolutionary trends in Vif (slope = -0.00024, *p* < 0.0001), suggesting an adaptation to the host environment, possibly aimed at maintaining overall sequence conservation close to its consensus state for optimal fitness.

Despite being computational, our analysis of over 50,000 sequences of global circulating strains from 1998 to 2021 provides a snapshot of how complex selection pressures influence Vif's mutational patterns. While our study primarily focused on point mutation, without differentiating between different selection pressures, it predicted a temporal decline in nonsynonymous mutations and mutational frequency in Vif against a consensus sequence. This pattern suggests Vif's reversion to its ancestral form, a likely strategy to preserve replicative fitness.

Reversion tendency in Vif (back to wild type) was indicated via structural stability predictions, specifically focused on the functional motifs that interact with A3F and A3G. Furthermore, mutation-reversion tendencies in Vif were also inferred through temporal analyses of site-specific mutational frequencies, which also suggested the influence of HLA-restricted immune pressures on Vif's overall evolutionary trajectory. Positions that are conserved across different subtypes may be under functional constraints, possibly due to their critical interactions with host proteins. On the other hand, the more variable positions could be hotspots for mutations driven by host immune pressure, influencing Vif's overall evolutionary trajectory. While the evolutionary pattern of Vif does not necessarily align with the broader evolutionary shifts of HIV, the patterns are consistent with HIV-1 polymorphism reported in various studies. Thus, these insights from our study may be crucial for future anti-HIV-1 treatment targeting Vifs' antagonistic function.

## 5 Conclusion

Our study provides novel insights into the mutational and evolutionary landscape of HIV-1 Vif, laying the foundation for targeted HIV treatments. Utilizing a dataset of over 50,000 sequences, our in-silico analysis indicates Vif is currently under positive selection while gradually adapting to the host environment, thus maintaining a balance between immune pressure and viral fitness. The consistent pattern of global and temporal amino acid variability in Vif makes it a promising target for universal HIV vaccines. Further, the functional motif of Vif exhibited sequence conservation while mutational plasticity was also observed, presenting a promising avenue for therapeutic vaccines and small molecule inhibitors. In contrast, HLA-restricted polymorphic sites, as epitopes, can prime T cells against a wide range of antigenic peptides, thereby enhancing immunogenic responses. Further, this pilot study may pave the way for future research focusing on deep learning to capture the mutational dynamics, immunogenicity, and future evolutionary trends of Vif from large sequence datasets, which might potentially revolutionize the conventional treatment design.

## Declaration of generative AI and AI-assisted technologies

During the preparation of this work, the authors used GPT-4 in order to improve language and readability. After using this tool, the authors reviewed and edited the content as needed and take full responsibility for the content of the publication.

## Funding

This research did not receive any specific grant from funding agencies in the public, commercial, or not-for-profit sectors.

## Authors statement

The Authors declare that this manuscript is original, has not been published before, and is not currently being considered for publication elsewhere. The manuscript and revised manuscript have been read and approved by all authors, and there are no other persons who satisfied the criteria for authorship but are not listed.

## Supporting information

Supplementary Fig. SF1

Supplementary Tables (Supplementary Table ST1–3).

## CRediT authorship contribution statement

**Md Sakil Arman:** Writing – original draft, Visualization, Software, Methodology, Investigation, Formal analysis, Data curation. **Md Zafrul Hasan:** Writing – review & editing, Validation, Supervision, Resources, Funding acquisition, Conceptualization.

## Declaration of competing interest

The authors declare that they have no known competing financial interests or personal relationships that could have appeared to influence the work reported in this paper.

## Data Availability

Data will be made available on request.

## Appendix Supplementary materials

Supplementary Fig. SF1 **Global distribution of HIV-1 subtypes in WHO epidemic region**. Countries are color-coded according to WHO epidemic regions for comprehensive visualization, with the most prominent subtype overlaid on each country (i.e., the subtype with the highest sequence count). Supplementary Fig. SF1:

Supplenmatry Tables: Detailed information on Vif global sequence distribution with prominent subtypes and mutations (ST1). Selection pressure and immunological relevance of Vif residues (ST2) and stability of Vif residues upon dominant mutation (ST3). Supplenmatry Tables:

## References

[bib0001] Akari H., Fujita M., Kao S., Khan M.A., Shehu-Xhilaga M., Adachi A. (2004). High level expression of human immunodeficiency virus type-1 Vif inhibits viral infectivity by modulating proteolytic processing of the gag precursor at the p2/nucleocapsid processing site. J. Biol. Chem..

[bib0002] Batisse J., Guerrero S., Bernacchi S., Sleiman D., Gabus C., Darlix J.L. (2012). The role of Vif oligomerization and RNA chaperone activity in HIV-1 replication. Virus Res..

[bib0003] Brumme Z.L., Brumme C.J., Heckerman D., Korber B.T., Daniels M., Carlson J. (2007). Evidence of differential HLA class I-mediated viral evolution in functional and accessory/regulatory genes of HIV-1. PLoS Pathog..

[bib0004] Brumme, Z.L., John, M., Carlson, J.M., Brumme, C.J., Chan, D., Brockman, M.A., et al., 2009. HLA-Associated immune escape pathways in HIV-1 subtype B Gag, pol and Nef Proteins. Nixon DF, editor. PLoS One [Internet] 4 (8), e6687. 10.1371/journal.pone.000668. PMC2723923 19690614

[bib0005] Carlson J.M., Brumme Z.L., Rousseau C.M., Brumme C.J., Matthews P., Kadie C. (2008). Phylogenetic dependency networks: inferring patterns of CTL escape and codon covariation in HIV-1 gag. PLoS Comput Biol [Internet].

[bib0006] Carlson J.M., Brumme Z.L. (2008). HIV evolution in response to HLA-restricted CTL selection pressures: a population-based perspective. Microbes Infect [Internet].

[bib0007] Carlson J.M., Listgarten J., Pfeifer N., Tan V., Kadie C., Walker B.D. (2012). Widespread impact of HLA restriction on immune control and escape pathways of HIV-1. J. Virol..

[bib0008] Casartelli N., Guivel-Benhassine F., Bouziat R., Brandler S., Schwartz O., Moris A. (2010). The antiviral factor APOBEC3G improves CTL recognition of cultured HIV-infected T cells. J. Exp. Med..

[bib0009] Chen G., He Z., Wang T., Xu R., Yu X.F. (2009). A patch of positively charged amino acids surrounding the human immunodeficiency virus type 1 Vif SLVx4Yx9Y motif influences its interaction with APOBEC3G. J. Virol..

[bib0010] Chen Y., Lu H., Zhang N., Zhu Z., Wang S., Li M., Prem P.S. (2020). Predicting the impact of missense mutations on protein stability. PLoS Comput. Biol..

[bib0011] Cock P.J.A., Antao T., Chang J.T., Chapman B.A., Cox C.J., Dalke A. (2009). Biopython: freely available python tools for computational molecular biology and bioinformatics. Bioinformatics.

[bib0012] Crooks G.E., Hon G., Chandonia J.M., Brenner S.E. (2004). WebLogo: a sequence logo generator. Genome Res..

[bib0013] Dang Y., Davis R.W., York I.A., Zheng Y.H. (2010). Identification of 81 LGxGxxIxW 89 and 171 EDRW 174 domains from human immunodeficiency virus type 1 Vif that regulate APOBEC3G and APOBEC3F neutralizing activity. J. Virol..

[bib0014] Dang Y., Wang X., Zhou T., York I.A., Zheng Y. (2009). Identification of a novel WxSLVK motif in the N terminus of human immunodeficiency virus and simian immunodeficiency virus Vif that is critical for APOBEC3G and APOBEC3F neutralization. J Virol [Internet].

[bib0015] Dimitrov D., Kublin J.G., Ramsey S., Corey L (2015). Are clade specific HIV vaccines a necessity? An analysis based on mathematical models. EBioMedicine.

[bib0016] Dobson C.M., Šali A., Karplus M. (1998). Protein folding: a perspective from theory and experiment. Angew. Chem. Int. Ed..

[bib0017] Edgar R.C. (2004). MUSCLE: multiple sequence alignment with high accuracy and high throughput. Nucleic Acids Res..

[bib0018] Erdmann N., Du V.Y., Carlson J., Schaefer M., Jureka A., Sterrett S. (2015). HLA class-II associated HIV polymorphisms predict escape from CD4+ T cell responses. PLoS Pathog..

[bib0019] Greenwood E.J.D., Matheson N.J., Wals K., van den Boomen D.J.H., Antrobus R., Williamson J.C. (2016). Temporal proteomic analysis of HIV infection reveals remodelling of the host phosphoproteome by lentiviral Vif variants. eLife.

[bib0020] Harris R.S., Bishop K.N., Sheehy A.M., Craig H.M., Petersen-Mahrt S.K., Watt I.N. (2003). DNA deamination mediates innate immunity to retroviral infection. Cell.

[bib0021] Hasan Z., Carlson J.M., Gatanaga H., Le A.Q., Brumme C.J., Oka S. (2012). Minor contribution of HLA class I-associated selective pressure to the variability of HIV-1 accessory protein Vpu. Biochem. Biophys. Res. Commun..

[bib0022] Hasan Z., Hasan M., Ashik A.I., Mahtarin R., Newaj M.A., Nishat Z.S. (2020). Prediction of immune pressure on HIV-1 regulatory gene tat by human host through bioinformatics tools. J. Adv. Biotechnol. Exp. Ther..

[bib0023] Haynes B.F., Wiehe K., Borrow P., Saunders K.O., Korber B., Wagh K. (2023). Strategies for HIV-1 vaccines that induce broadly neutralizing antibodies. Nat. Rev. Immunol..

[bib0025] He Z., Zhang W., Chen G., Xu R., Yu X. (2008). Characterization of conserved motifs in HIV-1 Vif required for APOBEC3G and APOBEC3F interaction. J Mol Biol [Internet].

[bib0026] Herbeck J.T., Nickle D.C., Learn G.H., Gottlieb G.S., Curlin M.E., Heath L. (2006). Human immunodeficiency virus type 1 env evolves toward ancestral states upon transmission to a new host. J. Virol..

[bib0027] Hultquist J.F., Lengyel J.A., Refsland E.W., LaRue R.S., Lackey L., Brown W.L. (2011). Human and rhesus APOBEC3D, APOBEC3F, APOBEC3G, and APOBEC3H demonstrate a conserved capacity to restrict Vif-deficient HIV-1. J. Virol..

[bib0028] Hunter J.D. (2007). Matplotlib: a 2D graphics environment. Comput. Sci. Eng..

[bib0030] Hüttenhain R., Xu J., Burton L.A., Gordon D.E., Hultquist J.F., Johnson J.R. (2019). ARIH2 Is a Vif-dependent regulator of CUL5-mediated APOBEC3G degradation in HIV infection. Cell Host Microbe.

[bib0031] Jäger S., Cimermancic P., Gulbahce N., Johnson J.R., McGovern K.E., Clarke S.C. (2012). Global landscape of HIV-human protein complexes. Nature.

[bib0032] Jordahl K., den Bossche J.V., Fleischmann M., Wasserman J., McBride J. (2020). geopandas/geopandas: v0.8.1 (v0.8.1). Zenodo. J. Gerard.

[bib0033] Jost S., Altfeld M. (2012). Evasion from NK cell-mediated immune responses by HIV-1. Microbes Infect..

[bib72] Jumper, J., Evans, R., Pritzel, A., Green, T., Figurnov, M., Ronneberger, O., et al., 2021. Highly accurate protein structure prediction with AlphaFold. Nature [Internet] 596 (7873), 583–589. 10.1038/s41586-021-03819-22. PMC8371605 34265844

[bib0034] Korber B. (2002). Computational and Evolutionary Analysis of HIV Molecular Sequences.

[bib0035] Kumar S., Stecher G., Li M., Knyaz C., Tamura K., Battistuzzi F.U. (2018). MEGA X: molecular evolutionary genetics analysis across computing platforms. Mol. Biol. Evol..

[bib0036] Leslie A.J., Pfafferott K.J., Chetty P., Draenert R., Addo M.M., Feeney M. (2004). HIV evolution: CTL escape mutation and reversion after transmission. Nat. Med..

[bib0037] Li Y-L, Langley C.A., Azumaya C.M., Echeverria I., Chesarino N.M., Emerman M. (2023). The structural basis for HIV-1 Vif antagonism of human APOBEC3G. Nature [Internet].

[bib0038] Linchangco GV, Foley B, Leitner T (2022). Updated HIV-1 Consensus Sequences Change but Stay Within Similar Distance From Worldwide Samples. Front. Microbiol..

[bib0039] Mansky L.M., Temin H.M. (1995). Lower *in vivo* mutation rate of human immunodeficiency virus type 1 than that predicted from the fidelity of purified reverse transcriptase. J. Virol..

[bib0040] Marelli S., Williamson J.C., Protasio A.V., Naamati A., Greenwood E.J.D., Deane J.E. (2020). Antagonism of PP2A is an independent and conserved function of HIV-1 Vif and causes cell cycle arrest. eLife.

[bib0041] Matsui Y., Shindo K., Nagata K., Io K., Tada K., Iwai F. (2014). Defining HIV-1 Vif residues that interact with CBFβ by site-directed mutagenesis. Virology.

[bib0042] Mehle A., Thomas E.R., Rajendran K.S., Gabuzda D. (2006). A zinc-binding region in Vif binds Cul5 and determines cullin selection. J. Biol. Chem..

[bib0043] Mehle A., Wilson H., Zhang C., Brazier A.J., McPike M., Pery E. (2007). Identification of an APOBEC3G binding site in human immunodeficiency virus type 1 Vif and inhibitors of Vif-APOBEC3G binding. J. Virol..

[bib73] Mirdita M., Schütze K., Moriwaki Y., Heo L., Ovchinnikov S., Steinegger M. (2022). ColabFold: making protein folding accessible to all. Nat Methods [Internet].

[bib0044] Montanucci L., Capriotti E., Birolo G., Benevenuta S., Pancotti C., Lal D. (2022). DDGun: an untrained predictor of protein stability changes upon amino acid variants. Nucleic Acids Res..

[bib0045] Moore C.B., John M., James I.R., Christiansen F.T., Witt C.S., Mallal S.A. (2002). Evidence of HIV-1 adaptation to HLA-restricted immune responses at a population level. Science.

[bib0046] Mujib S., Liu J., Rahman A.K.M.N., Schwartz J.A., Bonner P., Yue F.Y. (2017). Comprehensive cross-clade characterization of antibody-mediated recognition, complement-mediated lysis, and cell-mediated cytotoxicity of HIV-1 envelope-specific antibodies toward eradication of the HIV-1 reservoir. J Virol..

[bib0047] Mwimanzi P., Hasan Z., Hassan R., Suzu S., Takiguchi M., Ueno T. (2011). Effects of naturally-arising HIV Nef mutations on cytotoxic T lymphocyte recognition and Nef's functionality in primary macrophages. Retrovirology.

[bib0048] Mwimanzi P., Hasan Z., Tokunaga M., Gatanaga H., Oka S., Ueno T. (2010). Naturally arising HIV-1 Nef variants conferring escape from cytotoxic T lymphocytes influence viral entry co-receptor expression and susceptibility to superinfection. Biochem. Biophys. Res. Commun..

[bib0049] Nakashima M., Ode H., Kawamura T., Kitamura S., Naganawa Y., Awazu H. (2016). Structural insights into HIV-1 Vif-APOBEC3F interaction. J. Virol..

[bib0050] Oberste M.S., Gonda M.A. (1992). Conservation of amino-acid sequence motifs in lentivirus Vif proteins. Virus Genes.

[bib0051] O'Donnell T.J., Rubinsteyn A., Bonsack M., Riemer A.B., Laserson U., Hammerbacher J. (2018). MHCflurry: open-source class I MHC binding affinity prediction. Cell Syst..

[bib0052] O'Donnell T.J., Rubinsteyn A., Laserson U. (2020). MHCflurry 2.0: improved pan-allele prediction of MHC class I-presented peptides by incorporating antigen processing. Cell Syst..

[bib0053] Ooms M., Brayton B., Letko M., Maio S.M., Pilcher C.D., Hecht F.M. (2013). HIV-1 Vif adaptation to human APOBEC3H haplotypes. Cell Host Microbe.

[bib0054] Ooms M., Letko M., Binka M., Simon V. (2013). The resistance of human APOBEC3H to HIV-1 NL4-3 molecular clone is determined by a single amino acid in Vif. PLoS One.

[bib0055] Pery E., Rajendran K.S., Brazier A.J., Gabuzda D. (2009). Regulation of APOBEC3 proteins by a novel YXXL motif in human immunodeficiency virus type 1 vif and simian immunodeficiency virus SIVagm vif. J Virol [Internet].

[bib0056] Ragonnet-Cronin M., Aris-Brosou S., Joanisse I., Merks H., Vallee D., Caminiti K. (2012). Adaptive evolution of HIV at HLA epitopes is associated with ethnicity in Canada. PLoS One.

[bib0057] Refsland E.W., Hultquist J.F., Luengas E.M., Ikeda T., Shaban N.M., Law E.K. (2014). Natural polymorphisms in human APOBEC3H and HIV-1 Vif combine in primary T lymphocytes to affect viral G-to-A mutation levels and infectivity. PLoS Genet.

[bib0058] Robertson D.L., Anderson J.P., Bradac J.A., Carr J.K., Foley B., Funkhouser R.K. (2000). HIV-1 nomenclature proposal. Science.

[bib0059] Rodrigues C.H.M., Pires D.E.V., Ascher D.B. (2021). DynaMut2: assessing changes in stability and flexibility upon single and multiple point missense mutations. Protein Sci..

[bib0060] Russell R.A., Smith J., Barr R., Bhattacharyya D., Pathak V.K. (2009). Distinct domains within APOBEC3G and APOBEC3F interact with separate regions of human immunodeficiency virus type 1 Vif. J. Virol..

[bib0061] Salamango D.J., Ikeda T., Moghadasi S.A., Wang J., McCann J.L., Serebrenik A.A. (2019). HIV-1 Vif triggers cell cycle arrest by degrading cellular PPP2R5 phospho-regulators. Cell Rep..

[bib0062] Santoro M.M., Perno C.F. (2013). HIV-1 genetic variability and clinical implications. ISRN. Microbiol..

[bib0063] Sewell A.K., Price D.A., Oxenius A., Kelleher A.D., Phillips R.E. (2000). Cytotoxic T lymphocyte responses to human immunodeficiency virus: control and escape. Stem Cells.

[bib0064] Sheehy A.M., Gaddis N.C., Choi J.D., Malim M.H. (2002). Isolation of a human gene that inhibits HIV-1 infection and is suppressed by the viral Vif protein. Nature Nature.

[bib0065] Wang H., Liu B., Liu X., Li Z., Yu X.F., Zhang W. (2014). Identification of HIV-1 Vif regions required for CBF-β interaction and APOBEC3 suppression. PLoS One.

[bib0066] Williams A., Menon S., Crowe M., Agarwal N., Biccler .J, Bbosa N. (2023). Geographic and Population Distributions of Human Immunodeficiency Virus (HIV)–1 and HIV-2 Circulating Subtypes: A Systematic Literature Review and Meta-analysis (2010–2021). J Infect Dis [Internet].

[bib0067] Yu X., Yu Y., Liu B., Luo K., Kong W., Mao P. (2003). Induction of APOBEC3G ubiquitination and degradation by an HIV-1 Vif-Cul5-SCF complex. Science.

[bib0068] Yu Y., Xiao Z., Ehrlich E.S., Yu X., Yu X. (2004). Selective assembly of HIV-1 Vif-Cul5-ElonginB-ElonginC E3 ubiquitin ligase complex through a novel SOCS box and upstream cysteines. Genes Dev [Internet].

[bib0069] Zhang W., Du J., Evans S.L., Yu Y., Yu X.F. (2012). T-cell differentiation factor CBF-β regulates HIV-1 Vif-mediated evasion of host restriction. Nature.

[bib0070] Zheng Y.H., Lovsin N., Peterlin B.M. (2005). Newly identified host factors modulate HIV replication. Immunol. Lett..

[bib0071] Zhou Y., Pan Q., Pires D.E .V, Rodrigues C.H.M., Ascher D.B. (2023). DDMut: predicting effects of mutations on protein stability using deep learning. Nucleic Acids Res..

