# Supplementary material for: A computational exploration of global and temporal dynamics of selection pressure on HIV-1 Vif polymorphism
Source: Virus Res. 2024 Jan 19;341:199323. doi: 10.1016/j.virusres.2024.199323 (PMC10831783; doi:10.1016/j.virusres.2024.199323)
Supplement: Supplementary Fig. SF1 — Global distribution of HIV-1 subtypes in WHO epidemic region. Countries are color-coded according to WHO epidemic regions for comprehensive visualization, with the most prominent subtype overlaid on each country (i.e., the subtype with the highest sequence count). [file mmc1.pdf]

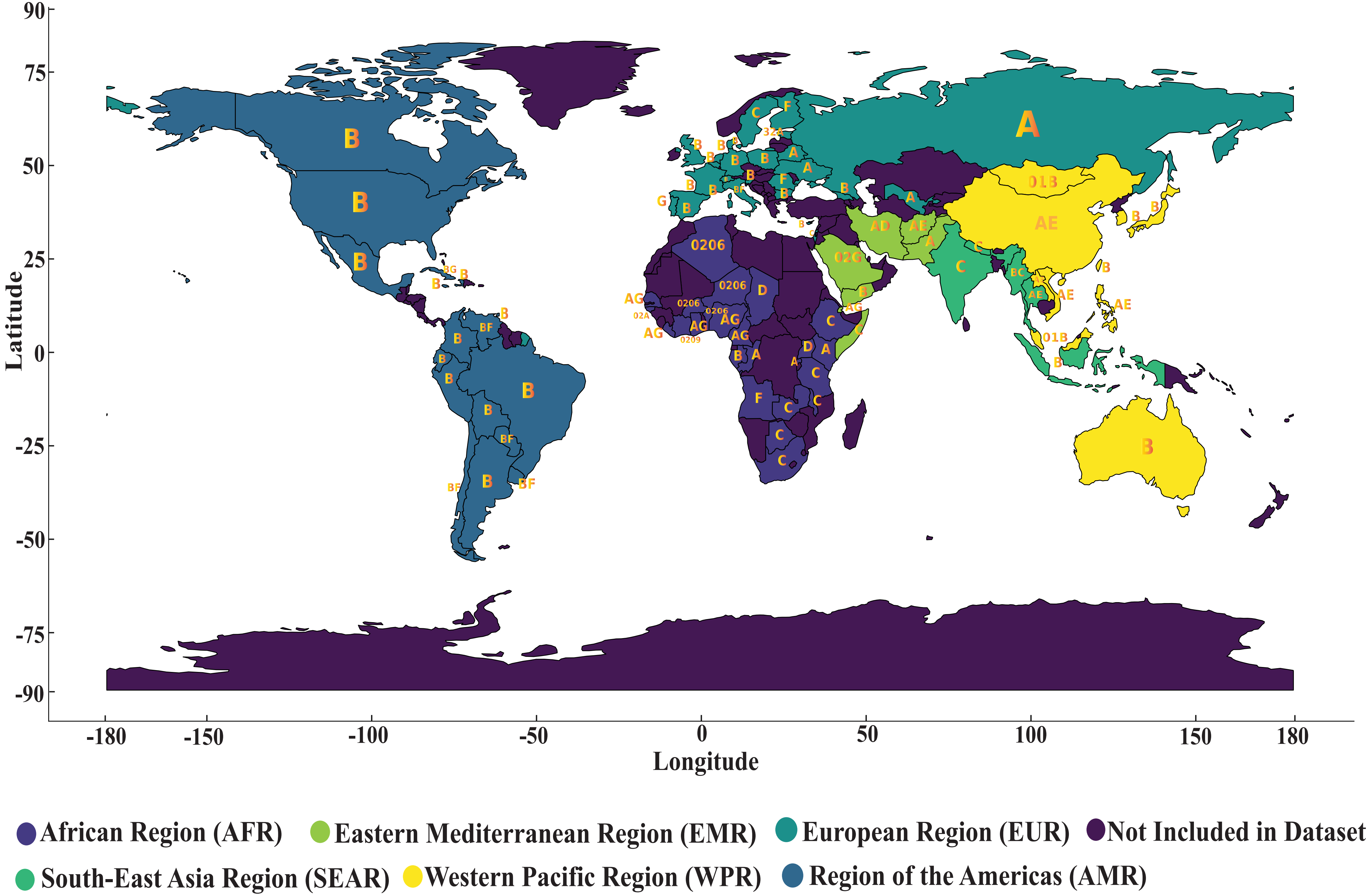
90
75
50
25
0
-25
-50
-75
-90
Latitude
-180
-150
-100
-50
0
50
100
150
180
Longitude
African Region (AFR)
Eastern Mediterranean Region (EMR)
European Region (EUR)
Not Included in Dataset
South-East Asia Region (SEAR)
Western Pacific Region (WPR)
Region of the Americas (AMR)
