# Supplementary material for: A computational exploration of global and temporal dynamics of selection pressure on HIV-1 Vif polymorphism
Source: Virus Res. 2024 Jan 19;341:199323. doi: 10.1016/j.virusres.2024.199323 (PMC10831783; doi:10.1016/j.virusres.2024.199323)
Supplement: Supplenmatry Tables: — Detailed information on Vif global sequence distribution with prominent subtypes and mutations (ST1). Selection pressure and immunological relevance of Vif residues (ST2) and stability of Vif residues upon dominant mutation (ST3). [file mmc2.docx]

**Supplementary Table ST1: Regional Distribution of Vif Sequences along with Predominant Subtypes and Key Mutations in Vif functional motif**

| Region | Country | Total  Sequence (n) | Prominent  Subtype | Prominent  Mutation | Country | Total  Sequences (n) | Prominent  Subtype | Prominent  Mutation |
| --- | --- | --- | --- | --- | --- | --- | --- | --- |
| **AFR** | Zambia | 334 | C | S116A | Ethiopia | 156 | C | Y110H |
|  | Senegal | 296 | AG | Y110H | Côte d'Ivoire | 91 | 0209 | I66V |
|  | Rwanda | 191 | A | Y110H | Congo | 402 | A | V65I |
|  | Nigeria | 441 | AG | I66V | Guinea-Bissau | 109 | 02A | I66V |
|  | Niger | 52 | 0206 | Y110H | Cameroon | 1521 | AG | H127Q |
|  | Tanzania | 1295 | C | Y110H | Chad | 42 | D | N22K |
|  | Uganda | 2032 | D | I66V | Botswana | 1107 | C | Y110H |
|  | Malawi | 228 | C | Y110H | Central African Republic | 98 | AE | - |
|  | Liberia | 13 | AG | Y110H | Benin | 7 | 0206 | N22K |
|  | Kenya | 2017 | A | I66V | Algeria | 1 | 0206 | N22K |
|  | South Africa | 5309 | C | Y110H | Burkina Faso | 7 | 0206 | N22K |
|  | Ghana | 252 | AG | I66V | Angola | 51 | F | N22K |
|  | Gabon | 24 | B | N22K |  |  |  |  |
| **EMR** | Iran | 7 | AD | R41K | Saudi Arabia | 26 | 02G | I66V |
|  | Yemen | 76 | B | T67K | Somalia | 1 | C | D61E |
|  | Djibouti | 2 | AG | Y44F | Afghanistan | 15 | AE | V65I |
|  | Pakistan | 166 | A | I66V |  |  |  |  |
| **EUR** | Romania | 7 | F | N22K | Georgia | 37 | B | I66V |
|  | Portugal | 30 | G | R63K | United Kingdom | 799 | B | N22K |
|  | Poland | 11 | B | D61E | Uzbekistan | 37 | A | I66V |
|  | Sweden | 785 | C | E117D | Cyprus | 1232 | B | I66V |
|  | Switzerland | 183 | B | N22K | Denmark | 247 | B | N22K |
|  | Netherlands | 287 | B | N22K | Bulgaria | 43 | B | R63K |
|  | Belarus | 12 | A | T67K | Germany | 2277 | B | N22K |
|  | Belgium | 321 | B | R63K | Estonia | 42 | 32A | I66V |
|  | Spain | 796 | B | R63K | Finland | 29 | F | Y44F |
|  | Ukraine | 160 | A | I66V | Israel | 73 | C | L109M |
|  | Slovenia | 20 | B | N22K | France | 963 | B | N22K |
|  | Russia | 380 | A | I66V | Italy | 99 | BF | Y110H |
| **AMR** | Colombia | 105 | B | N22K | Mexico | 363 | B | K122N |
|  | Canada | 213 | B | N22K | Brazil | 2863 | B | N22K |
|  | Cuba | 149 | BG | R77K | Haiti | 61 | B | N22K |
|  | Dominican Republic | 61 | B | N22K | Bolivia | 36 | B | N22K |
|  | Ecuador | 60 | B | R167K | Uruguay | 262 | BF | N22K |
|  | Jamaica | 90 | B | N22K | United States of America | 8193 | B | N22K |
|  | Paraguay | 82 | BF | N22K | Peru | 162 | B | N22K |
|  | Argentina | 1113 | B | N22K | Chile | 24 | BF | N22K |
|  | Venezuela | 16 | BF | N22K | Trinidad and Tobago | 56 | B | N22K |
| **SEAR** | Thailand | 2883 | AE | I128V | Myanmar | 220 | BC | E134D |
|  | Nepal | 56 | C | L109M | India | 481 | C | Y110H |
|  |  |  |  |  | Indonesia | 39 | B | D61E |
| **WPR** | Singapore | 11 | 01B | Y110H | Malaysia | 258 | 01B | R167K |
|  | Viet Nam | 93 | AE | R77K | Mongolia | 2 | 01B | D61E |
|  | Australia | 506 | B | R132S | Philippines | 98 | AE | N22K |
|  | China | 3487 | AE | D61E | South Korea | 1278 | B | Y110H |
|  | Hong Kong | 29 | AE | E117D | Japan | 957 | B | N22K |
|  | Laos | 89 | AE | R77K |  |  |  |  |

*AFR: African Region, AMR: Region of the Americas, EMR: Eastern Mediterranean Region, EUR: European Region, SEAR: South-East Asia Region, WPR: Western Pacific Region*

**Supplementary Table ST2: Impact of selection pressure on immunological relevance of Vif**

| Motif | Amino Acid Position | Selection Pressure | LANL database | | | | Mutation frequency (%) |
| --- | --- | --- | --- | --- | --- | --- | --- |
|  |  |  | *CD8+ Epitope*  *(n)* | *CD4+ epitope*  *(n)* | *Antibody Epitope*  *(n)* | *CD8+ escape mutation*  *(n)* |  |
|  | 1 | N/A | 4 | 0 | 0 | 0 | 0.06 |
|  | 2 | N/A | 4 | 0 | 0 | 0 | 1.4 |
|  | 3 | N/A | 6 | 0 | 0 | 0 | 0.59 |
|  | 4 | pos | 6 | 1 | 0 | 0 | 1.26 |
|  | 5 | N/A | 7 | 1 | 0 | 0 | 0.08 |
|  | 6 | pos | 7 | 1 | 0 | 0 | 0.25 |
|  | 7 | pos | 7 | 1 | 0 | 0 | 5.32 |
|  | 8 | N/A | 8 | 1 | 0 | 0 | 21.12 |
|  | 9 | pos | 8 | 1 | 0 | 0 | 2.46 |
|  | 10 | neg | 8 | 1 | 0 | 0 | 0.04 |
|  | 11 | N/A | 9 | 1 | 0 | 0 | 0.13 |
|  | 12 | neg | 7 | 1 | 0 | 0 | 0.45 |
|  | 13 | neg | 8 | 1 | 0 | 0 | 1.28 |
| F1 box | 14 | N/A | 8 | 1 | 0 | 0 | 0.29 |
|  | 15 | neg | 7 | 1 | 0 | 0 | 0.18 |
|  | 16 | N/A | 8 | 1 | 0 | 0 | 0.67 |
|  | 17 | pos | 32 | 1 | 0 | 0 | 14.22 |
|  | 18 | neg | 36 | 1 | 0 | 0 | 0.34 |
|  | 19 | pos | 36 | 1 | 0 | 1 | 21.81 |
|  | 20 | pos | 36 | 1 | 0 | 0 | 9.38 |
| A3F and A3G interactive residues | 21 | N/A | 36 | 1 | 0 | 0 | 0.08 |
|  | 22 | pos | 36 | 1 | 0 | 0 | 47.92 |
|  | 23 | neg | 46 | 1 | 0 | 0 | 0.62 |
|  | 24 | neg | 46 | 1 | 0 | 1 | 2.57 |
|  | 25 | neg | 49 | 1 | 0 | 0 | 0.83 |
|  | 26 | neg | 48 | 1 | 0 | 0 | 0.08 |
|  | 27 | neg | 23 | 0 | 0 | 0 | 14.9 |
|  | 28 | neg | 30 | 0 | 0 | 1 | 0.24 |
|  | 29 | pos | 28 | 0 | 0 | 0 | 12.49 |
|  | 30 | pos | 28 | 0 | 0 | 2 | 16.28 |
|  | 31 | pos | 49 | 0 | 0 | 6 | 49.76 |
|  | 32 | neg | 41 | 0 | 0 | 0 | 0.6 |
|  | 33 | pos | 44 | 0 | 0 | 11 | 32.58 |
|  | 34 | pos | 44 | 0 | 1 | 6 | 19.59 |
|  | 35 | neg | 44 | 0 | 1 | 6 | 5.47 |
|  | 36 | pos | 44 | 0 | 1 | 4 | 38.34 |
|  | 37 | pos | 34 | 0 | 1 | 3 | 42.53 |
|  | 38 | N/A | 34 | 0 | 1 | 0 | 0.16 |
| A3H interactive residues | 39 | pos | 34 | 0 | 1 | 6 | 48 |
| G box | 40 | neg | 12 | 0 | 1 | 0 | 0.2 |
|  | 41 | pos | 10 | 0 | 1 | 1 | 14.82 |
|  | 42 | neg | 9 | 0 | 1 | 0 | 0.73 |
|  | 43 | neg | 6 | 0 | 1 | 0 | 0.15 |
|  | 44 | pos | 6 | 0 | 1 | 0 | 11.96 |
|  | 45 | pos | 6 | 0 | 1 | 0 | 11.66 |
|  | 46 | neg | 7 | 0 | 1 | 0 | 11.11 |
|  | 47 | pos | 7 | 0 | 1 | 1 | 57.48 |
| A3H interactive residues | 48 | pos | 37 | 0 | 0 | 2 | 21.7 |
|  | 49 | neg | 37 | 0 | 0 | 0 | 0.19 |
|  | 50 | pos | 37 | 0 | 0 | 6 | 42.41 |
|  | 51 | pos | 35 | 0 | 0 | 3 | 50.23 |
|  | 52 | neg | 35 | 0 | 0 | 0 | 2.03 |
|  | 53 | neg | 35 | 0 | 0 | 0 | 0.38 |
|  | 54 | neg | 38 | 0 | 0 | 1 | 0.71 |
| FG box | 55 | neg | 38 | 0 | 0 | 1 | 1.85 |
|  | 56 | neg | 39 | 0 | 0 | 2 | 2.62 |
|  | 57 | neg | 57 | 0 | 0 | 2 | 0.66 |
|  | 58 | neg | 26 | 0 | 0 | 0 | 0.29 |
|  | 59 | neg | 25 | 0 | 0 | 1 | 6.35 |
|  | 60 | neg | 25 | 0 | 0 | 0 | 9.82 |
|  | 61 | pos | 33 | 0 | 0 | 2 | 43.02 |
|  | 62 | neg | 34 | 0 | 0 | 1 | 6.43 |
|  | 63 | pos | 34 | 0 | 0 | 3 | 52.82 |
|  | 64 | neg | 33 | 0 | 0 | 0 | 5.21 |
|  | 65 | pos | 33 | 1 | 0 | 0 | 10.45 |
|  | 66 | pos | 31 | 1 | 0 | 2 | 35.37 |
|  | 67 | pos | 15 | 1 | 0 | 2 | 56.01 |
|  | 68 | neg | 15 | 1 | 0 | 0 | 0.55 |
|  | 69 | neg | 16 | 1 | 0 | 0 | 0.32 |
|  | 70 | pos | 10 | 1 | 0 | 0 | 0.12 |
|  | 71 | neg | 10 | 1 | 0 | 0 | 0.67 |
|  | 72 | neg | 10 | 1 | 0 | 0 | 0.34 |
|  | 73 | pos | 11 | 1 | 0 | 1 | 38.56 |
| F2 box | 74 | pos | 11 | 1 | 0 | 2 | 8.44 |
|  | 75 | neg | 11 | 1 | 0 | 0 | 0.06 |
|  | 76 | neg | 11 | 1 | 0 | 0 | 0.45 |
|  | 77 | pos | 11 | 0 | 0 | 0 | 23.04 |
|  | 78 | pos | 13 | 0 | 0 | 2 | 22.67 |
|  | 79 | N/A | 39 | 0 | 0 | 0 | 0.14 |
|  | 80 | neg | 39 | 0 | 0 | 2 | 11.35 |
|  | 81 | neg | 40 | 1 | 0 | 0 | 0.69 |
|  | 82 | neg | 36 | 1 | 0 | 0 | 0.15 |
|  | 83 | pos | 36 | 1 | 0 | 2 | 50.95 |
| CBF Beta | 84 | neg | 35 | 1 | 0 | 0 | 0.17 |
|  | 85 | pos | 38 | 1 | 0 | 3 | 5.98 |
|  | 86 | neg | 38 | 1 | 0 | 2 | 1.05 |
|  | 87 | pos | 41 | 1 | 0 | 1 | 2.9 |
|  | 88 | neg | 13 | 1 | 0 | 2 | 0.81 |
|  | 89 | pos | 15 | 1 | 0 | 0 | 0.09 |
|  | 90 | neg | 11 | 1 | 0 | 0 | 5.77 |
|  | 91 | pos | 9 | 1 | 0 | 2 | 62.34 |
|  | 92 | pos | 9 | 1 | 0 | 5 | 48.51 |
|  | 93 | pos | 10 | 1 | 0 | 3 | 30.11 |
|  | 94 | neg | 12 | 1 | 0 | 0 | 0.54 |
|  | 95 | pos | 9 | 1 | 0 | 2 | 25.36 |
|  | 96 | neg | 9 | 1 | 0 | 0 | 0.49 |
|  | 97 | neg | 10 | 0 | 0 | 0 | 0.21 |
|  | 98 | pos | 10 | 0 | 0 | 1 | 34.71 |
|  | 99 | neg | 10 | 0 | 0 | 3 | 8.72 |
|  | 100 | neg | 9 | 0 | 0 | 0 | 0.04 |
|  | 101 | pos | 21 | 0 | 0 | 7 | 53.13 |
| CBF Beta | 102 | neg | 33 | 0 | 0 | 5 | 7.07 |
|  | 103 | neg | 29 | 0 | 0 | 1 | 0.52 |
|  | 104 | neg | 28 | 0 | 0 | 5 | 0.22 |
|  | 105 | neg | 29 | 0 | 0 | 2 | 13.86 |
|  | 106 | neg | 31 | 0 | 0 | 0 | 0.58 |
|  | 107 | neg | 30 | 0 | 0 | 1 | 0.18 |
| Zinc finger region | 108 | neg | 30 | 0 | 0 | 0 | 0.06 |
|  | 109 | pos | 31 | 0 | 0 | 7 | 37.21 |
|  | 110 | pos | 21 | 0 | 0 | 7 | 51.27 |
|  | 111 | neg | 17 | 0 | 0 | 0 | 2.48 |
|  | 112 | pos | 5 | 0 | 0 | 0 | 0.18 |
|  | 113 | neg | 8 | 0 | 0 | 0 | 8.76 |
|  | 114 | neg | 9 | 0 | 0 | 0 | 0.14 |
|  | 115 | N/A | 9 | 0 | 0 | 0 | 0.17 |
|  | 116 | pos | 9 | 0 | 0 | 1 | 28.23 |
|  | 117 | pos | 9 | 0 | 0 | 4 | 45.77 |
|  | 118 | pos | 9 | 0 | 0 | 0 | 3.42 |
|  | 119 | neg | 12 | 0 | 0 | 0 | 0.18 |
| Cul5 interacting residues | 120 | pos | 12 | 0 | 0 | 1 | 1.27 |
|  | 121 | neg | 13 | 0 | 0 | 1 | 0.47 |
|  | 122 | pos | 12 | 0 | 0 | 8 | 45.82 |
|  | 123 | pos | 12 | 0 | 0 | 0 | 2.62 |
| Zinc finger region | 124 | pos | 9 | 0 | 0 | 1 | 11.43 |
|  | 125 | pos | 8 | 0 | 0 | 1 | 5.73 |
|  | 126 | neg | 8 | 0 | 0 | 0 | 0.81 |
|  | 127 | pos | 10 | 0 | 0 | 0 | 52.13 |
|  | 128 | pos | 7 | 0 | 0 | 0 | 50.69 |
|  | 129 | pos | 7 | 0 | 0 | 1 | 4.24 |
|  | 130 | pos | 6 | 0 | 0 | 0 | 46.68 |
|  | 131 | pos | 3 | 0 | 0 | 0 | 14.31 |
|  | 132 | pos | 3 | 0 | 0 | 0 | 25.81 |
|  | 133 | neg | 3 | 0 | 0 | 0 | 0.16 |
|  | 134 | pos | 3 | 0 | 0 | 0 | 38.16 |
|  | 135 | neg | 3 | 0 | 0 | 0 | 2.42 |
|  | 136 | pos | 0 | 0 | 0 | 0 | 23.24 |
|  | 137 | pos | 0 | 0 | 0 | 0 | 16.84 |
|  | 138 | neg | 0 | 0 | 0 | 0 | 0.14 |
|  | 139 | neg | 0 | 0 | 0 | 0 | 0.19 |
|  | 140 | neg | 0 | 0 | 0 | 0 | 11.01 |
|  | 141 | pos | 0 | 0 | 0 | 0 | 6.47 |
|  | 142 | pos | 0 | 0 | 0 | 0 | 0.45 |
|  | 143 | neg | 0 | 0 | 0 | 0 | 1.22 |
| BC box | 144 | neg | 0 | 2 | 0 | 0 | 1.49 |
|  | 145 | neg | 0 | 2 | 0 | 0 | 0.33 |
|  | 146 | neg | 0 | 2 | 0 | 0 | 0.31 |
|  | 147 | neg | 0 | 2 | 0 | 0 | 0.64 |
|  | 148 | neg | 0 | 2 | 0 | 0 | 0.14 |
|  | 149 | pos | 5 | 2 | 0 | 1 | 0.27 |
|  | 150 | neg | 5 | 2 | 0 | 1 | 0.52 |
|  | 151 | pos | 7 | 2 | 0 | 4 | 53.29 |
|  | 152 | pos | 7 | 2 | 0 | 0 | 2.89 |
|  | 153 | neg | 8 | 2 | 0 | 1 | 1.49 |
|  | 154 | pos | 8 | 2 | 0 | 3 | 44.46 |
|  | 155 | pos | 10 | 2 | 0 | 5 | 49.2 |
|  | 156 | pos | 10 | 2 | 0 | 1 | 5.75 |
|  | 157 | pos | 12 | 2 | 0 | 1 | 20.37 |
|  | 158 | pos | 28 | 2 | 0 | 3 | 36.78 |
|  | 159 | pos | 28 | 0 | 0 | 8 | 55.53 |
|  | 160 | pos | 31 | 0 | 0 | 3 | 16.1 |
|  | 161 | neg | 32 | 0 | 0 | 0 | 0.18 |
|  | 162 | neg | 32 | 0 | 0 | 0 | 0.09 |
| PPLP motif | 163 | neg | 32 | 0 | 0 | 0 | 0.62 |
|  | 164 | neg | 30 | 0 | 0 | 0 | 0.2 |
|  | 165 | pos | 31 | 0 | 0 | 0 | 0.28 |
|  | 166 | pos | 33 | 0 | 0 | 3 | 8.5 |
|  | 167 | pos | 31 | 0 | 0 | 1 | 62.44 |
|  | 168 | neg | 32 | 0 | 0 | 0 | 2.81 |
|  | 169 | neg | 12 | 0 | 0 | 0 | 0.12 |
|  | 170 | pos | 8 | 0 | 0 | 0 | 31.82 |
| F3 box | 171 | neg | 7 | 0 | 0 | 0 | 0.1 |
|  | 172 | neg | 5 | 0 | 0 | 0 | 0.46 |
|  | 173 | pos | 5 | 0 | 0 | 0 | 2.4 |
|  | 174 | pos | 5 | 0 | 0 | 0 | 0.12 |
|  | 175 | N/A | 3 | 0 | 0 | 0 | 1.1 |
|  | 176 | pos | 3 | 0 | 1 | 0 | 20.66 |
|  | 177 | pos | 2 | 0 | 1 | 0 | 3.73 |
|  | 178 | pos | 2 | 0 | 1 | 0 | 7.92 |
|  | 179 | pos | 2 | 0 | 1 | 0 | 16.86 |
|  | 180 | pos | 1 | 0 | 1 | 0 | 19.02 |
|  | 181 | pos | 1 | 0 | 1 | 0 | 46.99 |
|  | 182 | pos | 1 | 0 | 1 | 0 | 9.63 |
|  | 183 | pos | 1 | 0 | 1 | 0 | 27.61 |
|  | 184 | pos | 1 | 0 | 1 | 0 | 6.93 |
|  | 185 | pos | 0 | 0 | 1 | 0 | 22.47 |
|  | 186 | pos | 0 | 0 | 1 | 0 | 43.52 |
|  | 187 | pos | 0 | 0 | 1 | 0 | 19.77 |
|  | 188 | pos | 0 | 0 | 1 | 0 | 12.52 |
|  | 189 | pos | 0 | 0 | 1 | 0 | 6.6 |
|  | 190 | pos | 0 | 0 | 1 | 0 | 14.28 |
|  | 191 | neg | 0 | 0 | 1 | 0 | 0.43 |
|  | 192 | neg | 0 | 0 | 1 | 0 | 9.35 |

*Pos: positive selection (dn/ds>1), neg: Negative selection (dn/ds <1), N/A: Not available (e.g. division by 0)*

**Supplementary Table ST3: Impact of Point mutation on functional Motif of Vif**

| **Motif** | **Position** | **Wild Type** | **Dominating**  **Mutation** | **ΔΔG ^stability^ (Kcal/mol)** | | | | **Prediction** | **ΔΔG^stability^**(Reverse  Mutation) | **Prediction** |
| --- | --- | --- | --- | --- | --- | --- | --- | --- | --- | --- |
|  |  |  |  | **DynaMut2** | **DDMut** | **PremPS** | **DDGun3D** |  |  |  |
| **F1 box** | 14 | D (99.71%) | Y (0.09%) | 0.59 | 0.61 | -0.41 | 0.2 | Fav | -0.56 | N/F |
|  | 15 | R (99.82%) | G (0.17%) | -0.78 | 0.21 | -0.54 | -0.5 | N/F | 0.6 | Fav |
|  | 16 | M (99.33%) | R (0.17%) | 0.68 | 0.2 | -0.97 | -0.3 | N/F | -0.1 | N/F |
|  | 17 | R (85.78%) | K (14.05%) | -1.91 | -0.29 | -0.75 | -0.8 | N/F | 0.73 | Fav |
| **F2 box** | 74 | T (91.56%) | P (5.86%) | 0.06 | -1.11 | -0.39 | -0.6 | N/F | 1.2 | Fav |
|  | 75 | G (99.94%) | E (0.03%) | -0.09 | 0.4 | -1.25 | -0.6 | N/F | -0.33 | N/F |
|  | 76 | E (99.55%) | D (0.27%) | 0.08 | 0.27 | -0.28 | -0.1 | N/F | -0.29 | N/F |
|  | 77 | R (76.96%) | K (21.52%) | -0.19 | 0.14 | -0.39 | -0.5 | N/F | 0.6 | Fav |
|  | 78 | D (77.33%) | E (18.73%) | -0.06 | 0.11 | -0.05 | 0 | N/F | 0.03 | Fav |
|  | 79 | W (99.86%) | R (0.10%) | 0.18 | -0.18 | -0.66 | -1.8 | N/F | 0.48 | Fav |
| **F3 box** | 171 | E (99.90%) | G (0.04%) | -0.72 | -1.07 | -1.02 | -0.8 | N/F | 0.55 | Fav |
|  | 172 | D (99.54%) | N (0.34%) | -0.55 | -0.03 | -0.63 | -0.3 | N/F | -0.32 | N/F |
|  | 173 | R (97.60%) | K (2.06%) | -0.07 | -0.07 | -0.38 | -0.3 | N/F | 0.07 | Fav |
|  | 174 | W (99.88%) | R (0.07%) | 0.21 | 0.35 | -1.37 | -1.8 | N/F | 0.42 | Fav |
|  | 175 | N (98.90%) | S (0.53%) | 0.32 | 0.02 | -0.45 | -0.2 | N/F | 0.03 | Fav |
| **G box** | 40 | Y (99.80%) | F (0.10%) | -0.38 | 0.27 | -0.72 | -1 | N/F | -0.09 | N/F |
|  | 41 | R (85.18%) | K (14.20%) | -1.83 | -0.38 | -0.3 | -0.8 | N/F | 0.49 | Fav |
|  | 42 | H (99.27%) | P (0.25%) | 0.07 | -0.33 | -0.73 | -1.7 | N/F | 0.58 | Fav |
|  | 43 | H (99.85%) | P (0.05%) | 0.18 | 0.56 | -0.68 | -1.6 | N/F | -0.7 | N/F |
|  | 44 | Y (88.04%) | F (11.64%) | -0.34 | 0.05 | -0.06 | -0.4 | N/F | 0.08 | Fav |
| **FG box** | 55 | V (98.15%) | I (1.59%) | -0.58 | 0.86 | 0.11 | -0.5 | N/F | -0.94 | N/F |
|  | 56 | H (97.38%) | Y (1.52%) | 0.4 | 1.81 | -0.15 | 1 | Fav | -1.27 | N/F |
|  | 57 | I (99.34%) | V (0.56%) | -1.15 | 0.11 | -0.96 | -1.3 | N/F | -0.46 | N/F |
|  | 58 | P (99.71%) | L (0.17%) | 0 | 0.73 | -0.53 | -0.6 | N/F | -1.04 | N/F |
|  | 59 | L (93.65%) | I (3.70%) | -0.4 | -0.62 | -0.51 | -0.9 | N/F | 0.44 | Fav |
|  | 60 | G (90.18%) | E (6.06%) | 0.05 | 0.06 | -0.9 | -0.4 | N/F | 0.01 | Fav |
|  | 61 | D (56.98%) | E (37.37%) | -0.55 | 0.22 | -0.09 | 0 | N/F | -0.27 | N/F |
|  | 62 | A (93.57%) | D (2.20%) | -0.83 | -1.89 | -1.47 | -0.9 | N/F | 1.87 | Fav |
|  | 63 | R (47.18%) | K (38.09%) | -1.97 | -0.23 | 0.21 | -0.6 | N/F | 0.4 | Fav |
|  | 64 | L (94.79%) | I (3.83%) | -1.56 | -0.75 | -0.92 | -0.6 | N/F | 0.64 | Fav |
|  | 65 | V (89.55%) | I (9.61%) | -0.28 | -0.57 | -0.21 | -0.3 | N/F | 0.79 | Fav |
|  | 66 | I (64.63%) | V (35.18%) | -0.99 | 0.12 | -0.04 | -0.3 | N/F | -0.44 | N/F |
|  | 67 | T (43.99%) | K (24.83%) | -0.25 | -0.55 | -1.55 | -1.4 | N/F | 1.07 | Fav |
|  | 68 | T (99.45%) | A (0.39%) | -0.6 | -0.28 | -1.68 | -0.7 | N/F | 0.94 | Fav |
|  | 69 | Y (99.68%) | F (0.22%) | -0.84 | 0.81 | -0.79 | -0.8 | N/F | -0.79 | N/F |
|  | 70 | W (99.88%) | R (0.06%) | 0.17 | -0.61 | -1.29 | -3.3 | N/F | 1.28 | Fav |
|  | 71 | G (99.33%) | N (0.42%) | -0.31 | 0.21 | -1.13 | -0.5 | N/F | 0.56 | Fav |
|  | 72 | L (99.66%) | V (0.08%) | -1.04 | 0.55 | -0.91 | -1.4 | N/F | -0.68 | N/F |
| **A3F and A3G interactive residues** | 21 | W (99.92%) | C (0.03%) | -1.26 | -1.72 | -1.84 | -3.4 | N/F | 1.15 | Fav |
|  | 22 | N (52.08%) | K (38.56%) | -0.06 | 1.04 | -0.33 | -0.3 | N/F | -0.51 | N/F |
|  | 23 | S (99.38%) | G (0.34%) | -0.31 | -0.46 | -0.89 | -0.3 | N/F | 0.49 | Fav |
|  | 24 | L (97.43%) | I (2.35%) | -0.33 | -0.48 | -0.86 | -0.6 | N/F | 0.6 | Fav |
|  | 25 | V (99.17%) | I (0.78%) | -0.28 | 0.66 | -0.25 | -0.4 | N/F | -0.64 | N/F |
|  | 26 | K (99.92%) | R (0.05%) | -0.18 | 0.01 | -0.49 | 0.5 | N/F | 0.24 | Fav |
| **A3H interactive residues** | 39 | F (52.00%) | V (17.26%) | -1.06 | -0.51 | -0.43 | -0.3 | N/F | 0.64 | Fav |
|  | 48 | H (78.30%) | N (21.21%) | 0.07 | -0.09 | -0.25 | -0.6 | N/F | 0.47 | Fav |
| **CBF Beta** | 84 | G (99.83%) | E (0.07%) | -1.62 | 0.09 | -1.79 | -1.1 | N/F | -0.03 | N/F |
|  | 85 | V (94.02%) | A (4.34%) | -1.19 | -1.44 | -1.89 | -3 | N/F | 1.32 | Fav |
|  | 86 | S (98.95%) | A (0.92%) | -0.49 | -0.68 | -0.4 | 0.3 | N/F | 0.62 | Fav |
|  | 87 | I (97.10%) | V (1.85%) | -1.01 | -0.5 | -0.72 | -1.2 | N/F | 0.57 | Fav |
|  | 88 | E (99.19%) | V (0.26%) | 0 | 0.24 | -0.78 | 0.2 | Fav | -1.04 | N/F |
|  | 89 | W (99.91%) | R (0.04%) | -1.49 | -1.11 | -1.45 | -3.6 | N/F | 0.76 | Fav |
|  | 102 | L (92.93%) | I (1.81%) | -0.48 | -0.37 | -0.43 | -0.3 | N/F | 0.44 | Fav |
|  | 103 | A (99.48%) | T (0.42%) | -1.47 | -1.89 | -1.41 | -1.3 | N/F | 1.5 | Fav |
|  | 104 | D (99.78%) | G (0.10%) | -0.93 | -1.64 | -1.44 | -1.7 | N/F | 2.35 | Fav |
|  | 105 | Q (86.14%) | R (6.99%) | -0.25 | -0.46 | -0.3 | 0.1 | N/F | -0.63 | N/F |
|  | 106 | L (99.42%) | M (0.29%) | 0.19 | 0.11 | -0.51 | -1.1 | N/F | 0.24 | Fav |
|  | 107 | I (99.82%) | T (0.09%) | -1.6 | -2 | -2.87 | -4.4 | N/F | 1.32 | Fav |
| **Zinc finger region** | 108 | H (99.94%) | N (0.02%) | -1.61 | 0.15 | -1.4 | -2.2 | N/F | 0.4 | Fav |
|  | 109 | L (62.79%) | M (22.39%) | 0.11 | 0.04 | -0.17 | -0.2 | N/F | 0.1 | Fav |
|  | 110 | Y (48.73%) | H (43.82%) | -0.02 | -0.63 | -0.18 | -0.5 | N/F | 0.89 | Fav |
|  | 111 | Y (97.52%) | H (2.43%) | -1.22 | -0.88 | -1.1 | -1.9 | N/F | 1.31 | Fav |
|  | 112 | F (99.82%) | L (0.11%) | -1.92 | 0.37 | -1.69 | -2.4 | N/F | 0.97 | Fav |
|  | 113 | D (91.24%) | N (5.76%) | 0.08 | -0.27 | -0.43 | 0 | N/F | 0.36 | Fav |
|  | 114 | C (99.86%) | W (0.07%) | -0.87 | 0.37 | -1.14 | -2 | N/F | -0.39 | N/F |
|  | 115 | F (99.83%) | S (0.06%) | -2.83 | -0.85 | -2.22 | -4.1 | N/F | 0.63 | Fav |
|  | 116 | S (71.77%) | A (23.43%) | -0.1 | -0.41 | 0.05 | -0.2 | N/F | 0.57 | Fav |
|  | 117 | E (54.23%) | D (44.19%) | -0.29 | 0.07 | -0.02 | 0 | N/F | 0.09 | Fav |
|  | 118 | S (96.58%) | T (2.01%) | -0.13 | -0.09 | -0.06 | 0 | N/F | 0.21 | Fav |
|  | 119 | A (99.82%) | T (0.08%) | -0.87 | -1.38 | -1.34 | -0.9 | N/F | 1.3 | Fav |
|  | 120 | I (98.73%) | V (1.03%) | -0.17 | -0.25 | -0.33 | -0.7 | N/F | 0.56 | Fav |
|  | 121 | R (99.53%) | K (0.27%) | -0.13 | -0.02 | -0.41 | -0.4 | N/F | 0 | neutral |
|  | 122 | K (54.18%) | N (24.23%) | -0.99 | -0.93 | -0.22 | 0.1 | N/F | 0.95 | Fav |
|  | 123 | A (97.38%) | T (1.81%) | -0.99 | -0.47 | -1.13 | -1 | N/F | 0.43 | Fav |
|  | 124 | I (88.57%) | L (10.70%) | -0.13 | 0.03 | -0.22 | -0.3 | N/F | 0.09 | Fav |
|  | 125 | L (94.27%) | V (4.46%) | -0.1 | -0.03 | -0.52 | -0.6 | N/F | 0.14 | Fav |
|  | 126 | G (99.19%) | E (0.39%) | -0.24 | 0.05 | -1.07 | -0.4 | N/F | 0.04 | Fav |
|  | 127 | H (47.87%) | Q (25.81%) | 0.14 | 0.1 | -0.17 | -0.1 | N/F | -0.15 | N/F |
|  | 128 | I (49.31%) | V (24.82%) | 0.01 | 0.01 | -0.33 | -0.1 | N/F | 0.14 | Fav |
|  | 129 | V (95.76%) | I (2.98%) | -0.24 | -0.39 | 0.03 | -0.4 | N/F | 0.33 | Fav |
|  | 130 | S (53.32%) | R (24.37%) | -0.02 | 0.02 | -0.25 | 0.1 | N/F | 0 | neutral |
|  | 131 | P (85.69%) | H (5.27%) | -0.4 | -1.21 | -0.99 | -0.5 | N/F | 1.08 | Fav |
|  | 132 | R (74.19%) | S (18.58%) | -0.97 | -0.46 | -0.39 | -0.6 | N/F | 0.07 | Fav |
|  | 133 | C (99.84%) | W (0.06%) | -0.82 | -0.48 | -1.69 | -2.4 | N/F | 0.44 | Fav |
|  | 134 | E (61.84%) | D (27.73%) | -1.08 | -1.43 | -0.49 | -0.1 | N/F | 1.31 | Fav |
|  | 135 | Y (97.58%) | F (1.60%) | -0.79 | 0.61 | -0.9 | -0.8 | N/F | -0.42 | N/F |
|  | 136 | Q (76.76%) | P (18.67%) | -0.29 | 0.07 | -0.16 | 0 | N/F | 0.32 | Fav |
|  | 137 | A (83.16%) | S (9.73%) | -0.89 | -0.21 | -0.24 | -0.5 | N/F | 0.28 | Fav |
|  | 138 | G (99.86%) | R (0.09%) | -0.84 | -0.85 | -1.26 | -0.6 | N/F | 0.85 | Fav |
|  | 139 | H (99.81%) | L (0.07%) | 1.11 | -0.04 | -0.27 | -1.3 | N/F | 0.25 | Fav |
| **BC box** | 144 | S (98.51%) | T (0.80%) | -0.05 | 0.06 | -0.08 | 0.2 | N/F | 0.02 | Fav |
|  | 145 | L (99.67%) | P (0.08%) | -0.25 | -0.02 | -0.74 | -0.8 | N/F | -0.06 | N/F |
|  | 146 | Q (99.69%) | K (0.14%) | 0.19 | 0.02 | -0.28 | -0.4 | N/F | 0.07 | Fav |
|  | 147 | Y (99.36%) | F (0.34%) | -0.33 | 0.39 | -0.37 | -0.1 | N/F | -0.49 | N/F |
|  | 148 | L (99.86%) | W (0.07%) | -0.75 | 0.06 | -1.01 | -1.1 | N/F | -0.19 | N/F |
|  | 149 | A (99.73%) | T (0.16%) | -0.54 | -0.09 | -0.71 | -0.8 | N/F | 0.22 | Fav |
|  | 161 | P (99.82%) | L (0.11%) | -0.54 | -1.31 | -0.46 | -0.6 | N/F | 1.01 | Fav |
| **PPLP motif** | 162 | P (99.91%) | L (0.04%) | -0.4 | -0.41 | -0.56 | -0.5 | N/F | 0.3 | Fav |
|  | 163 | L (99.38%) | F (0.27%) | -1.43 | -0.85 | -1 | -0.8 | N/F | 1.02 | Fav |
|  | 164 | P (99.80%) | L (0.07%) | 0 | -0.26 | -1.17 | -0.3 | N/F | 0.03 | Fav |
|  | 165 | S (99.72%) | G (0.07%) | -0.66 | -2.2 | -1.43 | -0.8 | N/F | 1.85 | Fav |
|  | 166 | V (91.50%) | I (7.75%) | -0.67 | 0.15 | 0.02 | -0.4 | N/F | 0.13 | Fav |
|  | 167 | R (37.56%) | K (30.53%) | -1.36 | 0.06 | -0.14 | -0.5 | N/F | 0.08 | Fav |
|  | 168 | K (97.19%) | I (2.19%) | 1.09 | 0.15 | -0.4 | 0.3 | Fav | -0.64 | N/F |
|  | 169 | L (99.88%) | M (0.05%) | -0.23 | -0.58 | -0.62 | -1.3 | N/F | 0.07 | Fav |

*Fav: Favorable, N/F: Non-favorable*
